# Anti-Aging, Anti-Inflammatory, and Cytoprotective Properties of *Lactobacillus*- and Kombucha-Fermented *C. pepo* L. Peel and Pulp Extracts with Prototype Skin Toner Development

**DOI:** 10.3390/molecules30204082

**Published:** 2025-10-14

**Authors:** Aleksandra Ziemlewska, Zofia Nizioł-Łukaszewska, Martyna Zagórska-Dziok, Agnieszka Mokrzyńska, Witold Krupski, Magdalena Wójciak, Ireneusz Sowa

**Affiliations:** 1 Department of Technology of Cosmetic and Pharmaceutical Products, Medical College, University of Information Technology and Management in Rzeszow, Sucharskiego 2, 35-225 Rzeszow, Poland; zniziol@wsiz.edu.pl (Z.N.-Ł.); mzagorska@wsiz.edu.pl (M.Z.-D.); amokrzynska@wsiz.edu.pl (A.M.); 2 II Department of Medical Radiology, Medical University of Lublin, Aleje Raclawickie 1, 20-059 Lublin, Poland; witold.krupski@umlub.edu.pl; 3 Department of Analytical Chemistry, Medical University of Lublin, Aleje Raclawickie 1, 20-059 Lublin, Poland; magdalena.wojciak@umlub.edu.pl (M.W.); ireneusz.sowa@umlub.edu.pl (I.S.)

**Keywords:** *C. pepo* L., kombucha, *Lactobacillus* strains, cosmetic ingredients, anti-inflammatory properties, antiaging properties, skin cells, moisturizing toner

## Abstract

This study examined the cosmetic potential of extracts from the peel and pulp of fermented pumpkin (*Cucurbita pepo* L.). Fermentation was carried out using *Lactobacillus* strains (*L. plantarum*, *L. rhamnosus*, *L. fermentum*, and *L. paracasei*) and kombucha (SCOBY). Fermentation was carried out for 3 days (for lactic acid bacteria) and 10 and 20 days (for kombucha). The obtained products were analyzed by LC-MS for phytochemical composition and assessed for their antioxidant capacity (DPPH and ABTS assays) and ROS reduction in keratinocytes (HaCaT) and fibroblasts (HDF). The obtained ferments demonstrated cytoprotective effects (using Alamar Blue and Neutral Red assays). Both kombucha ferments and certain strains of *Lactobacillus* ferments demonstrated anti-aging effects (by inhibiting collagenase, elastase, and hyaluronidase) and anti-inflammatory effects (by significantly affecting IL-6 and IL-1β cytokine levels). A moisturizing skin toner containing the extracts and ferments was developed and tested for cytoprotective effects on HaCaT keratinocytes. The results confirm that fermented pumpkin peel and pulp extracts can be used as multifunctional cosmetic ingredients with the potential to provide antioxidant protection, anti-aging, and skin regeneration.

## 1. Introduction

Pumpkin (*Cucurbita pepo* L.) is an annual plant belonging to the *Cucurbitaceae* family, commonly used as a vegetable and valued for its rich nutritional content, including polysaccharides, carotenoids, amino acids, and essential minerals [1,2]. In addition to its traditional culinary and medicinal uses, recent studies highlight the potential of pumpkin peel and pulp as valuable sources of bioactive compounds that can be used in skin care and cosmetics [3].

Pumpkin peel, usually considered agro-waste, is particularly rich in phenolic acids (e.g., caffeic, ferulic, p-coumaric), flavonoids (e.g., quercetin, kaempferol), amino acids, and essential fatty acids. These components have strong antioxidant, anti-inflammatory, cytoprotective, and regenerative properties, making pumpkin peel extracts suitable for use in anti-aging and skin protection products [2,4]. The pulp, in addition to the polyphenols mentioned above, also contains high concentrations of carotenoids and vitamins C and E, which contribute to its antioxidant and photoprotective effects [2,5]. Therefore, pumpkin-derived ingredients are a balanced, multifunctional option for cosmetic preparations aimed at nourishing, improving hydration, and protecting the skin from harmful external factors.

Since oxidative stress is one of the main factors causing skin aging, the ability of pumpkin-derived compounds to counteract reactive oxygen species (ROS) is particularly important. Under physiological conditions, ROS are neutralized by endogenous antioxidant systems, but when this balance is disturbed, their excessive accumulation leads to oxidative stress. This condition disrupts collagen metabolism, promotes lipid peroxidation, and stimulates the release of pro-inflammatory cytokines, ultimately leading to deterioration of the skin structure and acceleration of the aging process [1,6,7,8,9].

In addition to the direct use of plant extracts in cosmetic formulations, fermentation products, including raw materials obtained with the participation of microorganisms such as lactic acid bacteria (LAB), are becoming increasingly important [10]. Examples of such ingredients include *Lactobacillus*/pumpkin ferment extract and *Lactobacillus*/pumpkin fruit ferment filtrate. These are products obtained by fermenting pumpkin fruit (*C. pepo* L.) with *Lactobacillus* bacteria, recognized as skin care ingredients. Lactic acid bacteria are characterized by safe metabolic activity in the environment of plant raw materials [11]. During fermentation, they convert available sugars into organic acids and other metabolites, which promote product preservation and improve its sensory and bioactive properties. An important aspect of fermentation is the increase in the antioxidant activity of the product [12]. Numerous studies have shown that fermented plant extracts have a higher ability to neutralize free radicals compared to unfermented extracts. This phenomenon is explained by the increased content of bioactive compounds, the release of phenols associated with cell wall structures, and increased enzymatic activity. Although lactic fermentation can occur spontaneously, in practice it is preferable to use starter cultures, which enable standardization and repeatability of the final product’s properties [13,14].

Fermentation using kombucha, a symbiotic culture of bacteria and yeast (SCOBY), is also gaining popularity. Traditionally, sweetened black or green tea is used as the fermentation medium, but literature suggests the possibility of using a wide range of plant materials [15,16]. Kombucha fermentation products contain, among other things, glucuronic acid, acetic acid, B vitamins, polyphenols, and enzymes that exhibit antioxidant, anti-inflammatory, skin-brightening, and regenerative properties [17,18].

The aim of this study was to investigate the potential of fermented extracts from pumpkin peel and pulp as new bioactive ingredients for cosmetic applications. To this end, extracts obtained by kombucha fermentation of varying duration and by bacterial fermentation with selected *Lactobacillus* strains were analyzed for their phytochemical composition using LC-MS. Their antioxidant activity was assessed using DPPH and ABTS tests, as well as in cell models through the modulation of reactive oxygen species in keratinocytes and fibroblasts. The cytoprotective properties of the extracts and ferments were additionally assessed by examining their effect on cell viability, proliferation, and metabolic functions. In addition, their potential anti-aging and anti-inflammatory effects were determined by measuring the activity of extracellular matrix-degrading enzymes and the secretion of pro-inflammatory cytokines. Finally, a model cosmetic preparation in the form of a moisturizing tonic containing selected fermented extracts was developed and subjected to a preliminary assessment of its cytoprotective activity in keratinocytes, providing initial verification of its usefulness in functional cosmetology.

## 2. Results

### 2.1. Chromatographic Analysis

**Figure 1:**
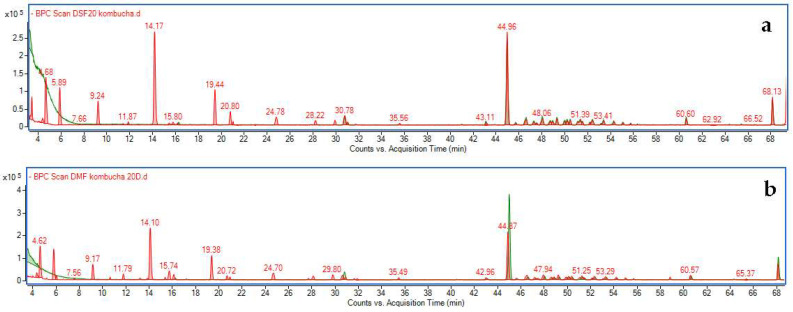
Base peak chromatogram of pumpkin extracts (green) overlapped with fermented extract after 20 days of fermentation (red); (**a**)—extract obtained from peel, (**b**)—extract obtained from pulp.

Fermented and unfermented extracts obtained from the peel and pulp of *Cucurbita pepo* L. (pumpkin) were analyzed using UPLC combined with DAD and TOF-MS detectors. Components were identified based on acquired UV-Vis spectra in the range of 200–600 nm and MS spectra recorded in the range of 100–1200 *m*/*z* at various ionization energies. When available, standards were used to confirm the identity of the compounds; otherwise, components were tentatively identified based on the recorded data. Table S1 (Supplementary Material) summarizes the mass data for the main peaks observed in the chromatograms. Examples of base peak chromatograms showing the overlaid traces for fermented and unfermented extracts from pumpkin skin and pulp are presented in Figure 1a and Figure 1b, respectively.

The profiles of the pulp and peel unfermented extracts were very similar and relatively poor (Figure S1), showing a strongly ionized, unidentified component (at a retention time of 44.9 min) that showed no absorption in the UV-Vis spectrum. Among phenolic compounds, only two components were identified—neochlorogenic acid and chlorogenic acid—with a total concentration of 4.83 ± 0.32 µg/g of dried peel extract and 3.63 ± 0.21 µg/g of dried pulp extract. In turn, the fermented extracts were rich in various components, including gallic acid and catechin derivatives, p-coumaroylquinic acids, chlorogenic acids, and flavonoids. Due to a lack of standards, different flavonoid derivatives were identified based on extracted ion chromatograms corresponding to the mass range specific for the respective aglycones (Figure S2), as well as their characteristic UV-Vis absorption spectra, which showed two absorption maxima in the ranges of 240–260 nm and 340–380 nm (Figure S3).

In general, fermented peel and pulp extracts showed a similar qualitative pattern, with the exception of a kaempferol derivative with [*m*/*z*-H]^−^ = 739, which was found only in the peel ferment. However, significant quantitative differences were observed. The differences in gallic acid, galloylquinic acid, and p-coumaroylquinic acid content between pulp and peel fermented extracts were only minor, and in both extracts, a significant increase was observed with longer fermentation periods. Gallocatechin and epigallocatechin levels were substantially higher in the pulp compared to the peel. During longer fermentation, their amounts did not increase in the pulp samples; however, similar to epicatechin, an increase was observed in the peel extract.

The total amount of apigenin, kaempferol, and quercetin derivatives after 10 days of fermentation was similar in both peel and pulp extracts, amounting to 76.2 ± 4.3 µg/g and 83.0 ± 5.2 µg/g, respectively. After prolonged fermentation, the total content increased significantly, especially in the pulp samples, reaching 95.7 ± 5.2 µg/g in the peel and 112.3 ± 7.8 µg/g in the pulp, representing an increase of 25.6% and 35.3%, respectively. Contrary to the trend observed for most flavonoid components, only kaempferol and quercetin 3-O-rutinoside showed no increase. Detailed results of the quantitative analysis are provided in Table 1.

Interestingly, no significant changes in the polyphenolic profile between fermented and unfermented extracts were observed when other microorganisms were used for fermentation (Figure S4), with the exception of chlorogenic acid in the peel samples. The concentration of chlorogenic acid in the skin extract fermented with *L. fermentum* and *L. plantarum* increased significantly compared to the unfermented peel extract (Figure S5). The total chlorogenic acid content reached 13.64 ± 0.75 µg/g for *L. fermentum* (~180% increase) and 5.49 ± 0.37 µg/g for *L. plantarum* (~13% increase), respectively. In other extracts, including the pulp extract, no changes or even decreases in chlorogenic acid content were observed.

As shown in our previous studies, the fermentation process is accompanied by an increase in gluconic acid as a result of the transformation of sucrose, which is added as one of the substrates. The efficiency of this process was very high in the case of kombucha fermentation (Figure S6).

### 2.2. Assessment of Skin Cell Viability Using the Alamar Blue and Neutral Red Assays

To evaluate the cytotoxic potential of pumpkin-based extracts and their ferments, two complementary in vitro assays were applied. The Alamar Blue test reflects mitochondrial metabolic activity, while the Neutral Red assay assesses lysosomal membrane integrity and function [19]. Both methods were performed using human keratinocytes (HaCaT) and dermal fibroblasts (HDF), which are widely used models in studies of skin cell viability.

**Figure 2:**
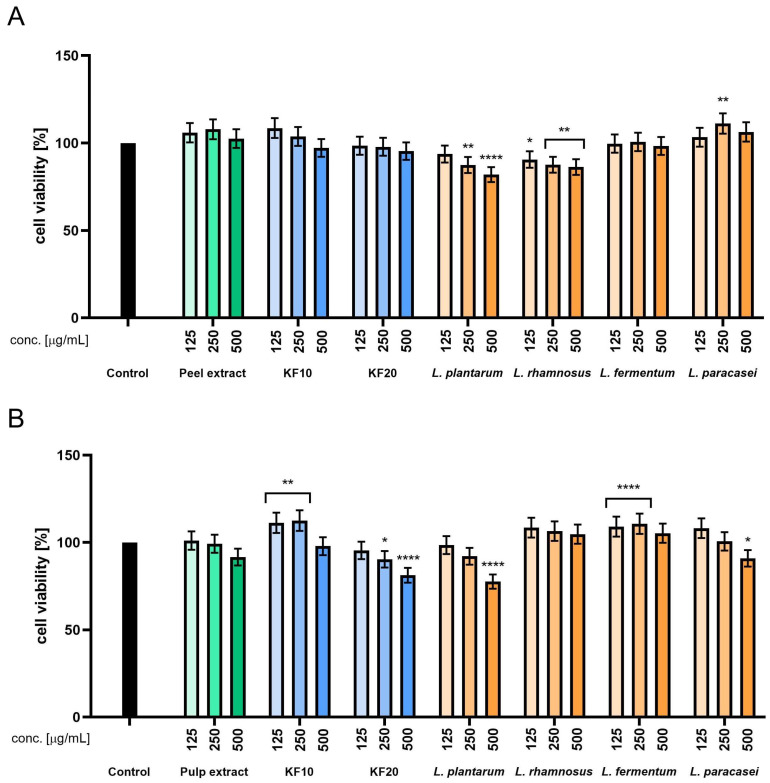
Assessment of resazurin reduction in keratinocytes (HaCaT) for *C. pepo* L. peel (**A**) and pulp (**B**) extract and ferments at the concentrations of 125, 250, and 500 µg/mL. Control cells without the addition of test samples, for which viability was assumed to be 100%. **** *p* < 0.0001, ** *p* < 0.01, * *p* < 0.05.

The Alamar Blue assay performed on HaCaT cells revealed that the pumpkin peel extract was non-cytotoxic across all tested concentrations (50–500 µg/mL), with cell viability values ranging from 100% to 105%. A mild stimulatory effect on mitochondrial activity was observed following 10-day kombucha fermentation (F10), particularly at 125–250 µg/mL, where viability exceeded 105%. Prolonged fermentation for 20 days (F20) resulted in slightly reduced metabolic activity, most notably at 500 µg/mL. Among the lactic acid bacteria (LAB) fermentations, *L. paracasei* demonstrated the most consistent and favorable effect on HaCaT cells, maintaining cell viability above 100% across all tested concentrations. Conversely, *L. plantarum* showed a concentration-dependent reduction in HaCaT cells viability, with a notable decrease observed at 500 µg/mL, indicating potential cytotoxicity at higher doses. The studies conducted on samples derived from pumpkin pulp demonstrated that kombucha fermentation for 10 days significantly enhanced cell viability, with F10-treated samples reaching approximately 115–118% at concentrations of 125–250 µg/mL. Ferments obtained with *L. rhamnosus*, *L. fermentum*, and *L. paracasei* displayed the most pronounced stimulatory effects, particularly at intermediate concentrations. As with peel-derived samples, *L. plantarum* exhibited cytotoxic tendencies at higher doses, reducing viability below 80% (Figure 2).

**Figure 3:**
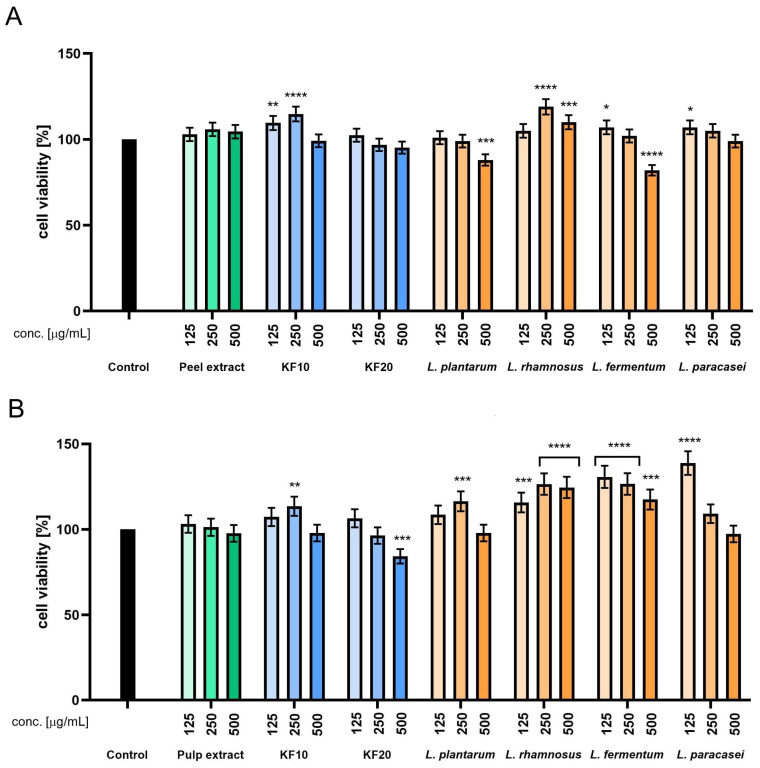
Assessment of resazurin reduction in fibroblasts (HDF) for *C. pepo* L. peel (**A**) and pulp (**B**) extract and ferments at the concentrations of 125, 250, and 500 µg/mL. Control cells without the addition of test samples, for which viability was assumed to be 100%. **** *p* < 0.0001, *** *p* < 0.001, ** *p* < 0.01, * *p* < 0.05.

In HDF cells, both peel and pulp extracts exerted neutral or slightly positive effects on mitochondrial activity. A moderate increase in viability was observed following exposure to the F10 peel ferment, with levels reaching up to 115%. Ferment prepared with *L. rhamnosus* significantly enhanced cell viability, reaching up to 119% at 500 µg/mL. Among pulp-derived samples, the most substantial increase was observed for the *L. paracasei* ferment at 50 µg/mL, which elevated metabolic activity to 135%. However, this effect decreased with increasing concentration, suggesting a dose-dependent plateau or potential inhibitory effect at higher levels. Ferments from *L. fermentum* and *L. rhamnosus* also increased fibroblast viability, reaching 130% and 126%, respectively (Figure 3).

**Figure 4:**
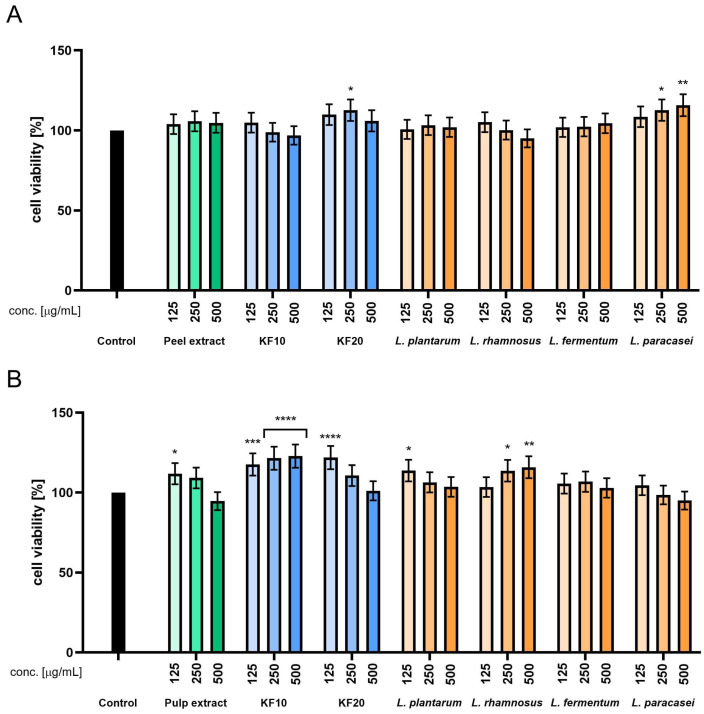
Assessment of neutral red uptake in keratinocytes (HaCaT) for *C. pepo* L. peel (**A**) and pulp (**B**) extract and ferments at the concentrations of 125, 250, and 500 µg/mL. Control cells without the addition of test samples, for which viability was assumed to be 100%. **** *p* < 0.0001, *** *p* = 0.0009, ** *p* < 0.01, * *p* < 0.05.

Neutral Red assay confirmed the absence of cytotoxicity in HaCaT cells exposed to both peel and pulp *C. pepo* L. extracts. A marked increase in lysosomal activity was observed following 20-day kombucha fermentation of peel samples, with viability reaching up to 112% at concentrations of 125–500 µg/mL. In contrast, F10-treated cells showed only minor fluctuations, with values remaining near baseline. Among the LAB ferments, *L. paracasei* produced a clear, dose-dependent increase in viability, reaching nearly 116% at the highest concentration. *L. fermentum* and *L. rhamnosus* also supported lysosomal activity, with values exceeding 105% at moderate and high doses (Figure 4).

For pulp-derived samples, fermentation with kombucha for both 10 and 20 days significantly enhanced the lysosomal activity of keratinocytes. Maximum viability was observed at 250 and 500 µg/mL of F10, reaching up to 123%. Although the F20 exhibited slightly lower biological activity compared to F10 ferment, treatment at a concentration of 125 µg/mL resulted in a notable increase in cell viability, reaching 122%. These results suggest that short-term fermentation of pumpkin pulp promotes the release of bioactive compounds capable of enhancing lysosomal membrane stability. LAB fermentations of the pulp also showed a stimulatory effect, particularly for *L. rhamnosus* (Figure 4).

**Figure 5:**
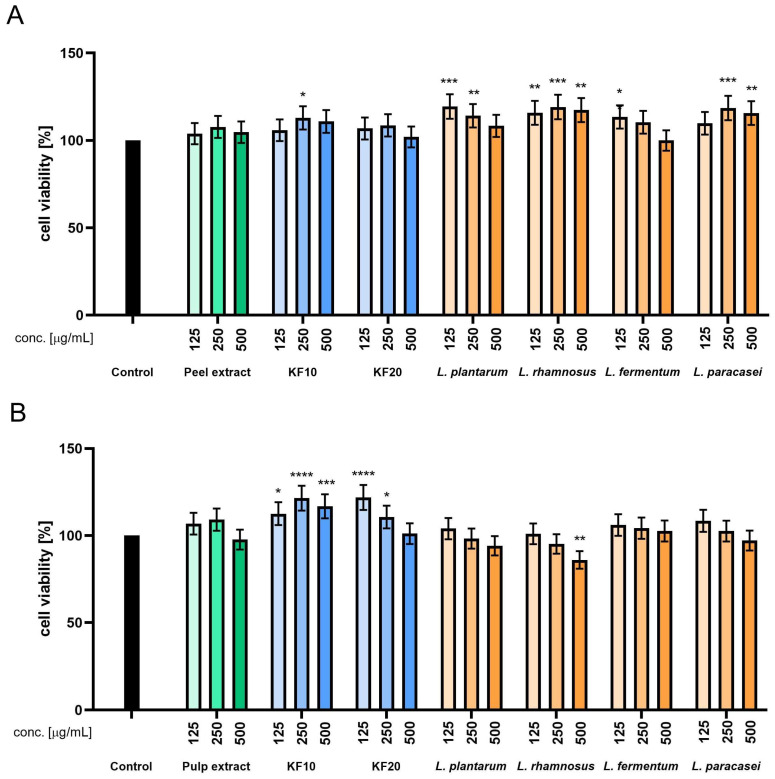
Assessment of neutral red uptake in fibroblasts (HDF) for *C. pepo* L. peel (**A**) and pulp (**B**) extract and ferments at the concentrations of 125, 250, and 500 µg/mL. Control cells without the addition of test samples, for which viability was assumed to be 100%. **** *p* < 0.0001, *** *p* < 0.001, ** *p* < 0.01, * *p* < 0.05.

The NR assay in HDF cells further confirmed the stimulatory potential of fermented pumpkin samples. Peel-derived ferments, especially those from *L. plantarum*, *L. rhamnosus*, and *L. paracasei*, significantly enhanced dye uptake (up to 119%), indicating improved lysosomal function and membrane stability. These effects were more prominent at intermediate concentrations, with some decline noted at 500 µg/mL (Figure 5).

Ferments from pumpkin pulp demonstrated the strongest overall lysosomal activity enhancement. F10 and F20 samples elevated viability above 120%. Ferments derived from lactic acid bacteria showed a viability-enhancing effect predominantly in the case of *L. fermentum* and *L. paracasei*, for which the concentration of 125 µg/mL was the most effective in stimulating cell viability. A dose-dependent trend was observed, with low to moderate concentrations stimulating lysosomal activity, while higher doses slightly reduced this response (Figure 5).

In the next stage of the study, the cytotoxicity of model cosmetic preparations—toners containing extracts from *C. pepo* L. peel and pulp, as well as their ferments—was assessed. The analyses were performed on human HaCaT keratinocytes, which are the main type of cells in the epidermis—the outermost layer of the skin that comes into direct contact with cosmetic preparations. The evaluation of the reaction of these cells makes it possible to determine the effect of the tested substances on the integrity and function of the skin’s protective barrier.

**Figure 6:**
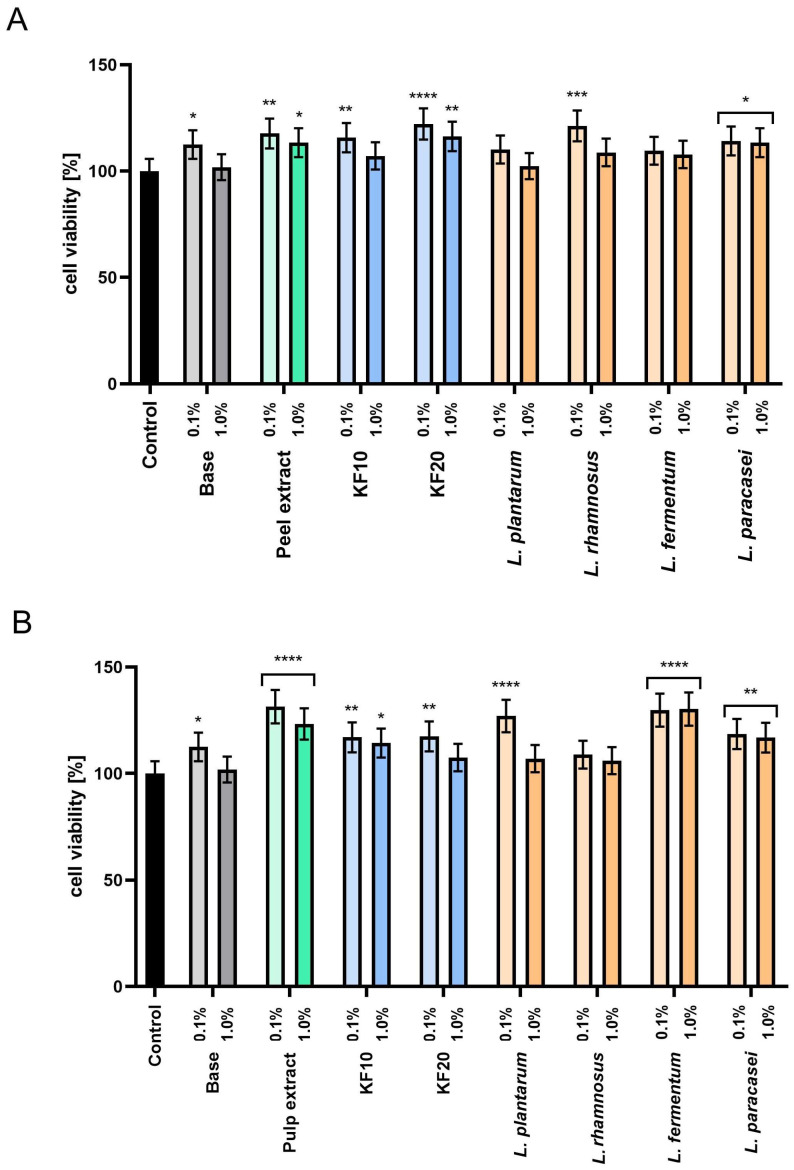
Assessment of resazurin reduction in keratinocytes (HaCaT) for moisturizing skin toners containing *C. pepo* L. peel (**A**) and pulp (**B**) extract and ferments at concentrations of 0.1 and 1.0%. Control cells without the addition of test samples, for which viability was assumed to be 100%. **** *p* < 0.0001, *** *p* = 0.0002, ** *p* < 0.01, * *p* < 0.05.

The tested compounds were introduced into toner formulations at a final concentration of 10% (*w*/*w*). For comparison, an analysis of the cytoprotective properties of the base tonic, which did not contain the tested extracts or ferments, was also performed. Cytotoxicity was assessed at two concentrations of the preparations: 1.0% and 0.1%. The results of the Alamar Blue test (Figure 6) showed that most samples were characterized by higher HaCaT cell proliferation compared to the base tonic. This effect was particularly pronounced at lower concentrations (0.1%), which significantly increased cell viability for most of the samples tested. For pumpkin peel extracts and ferments (Figure 6A), the most beneficial effect was observed for the KF20 and *L. rhamnosus* at a concentration of 0.1%, achieving 122.19% ± 0.11 and 121.29% ± 0.13 viability, respectively, compared to the control. In the case of pumpkin pulp (Figure 6B), in addition to the extract, *L. fermentum* showed a positive effect on proliferation at both concentrations tested

**Figure 7:**
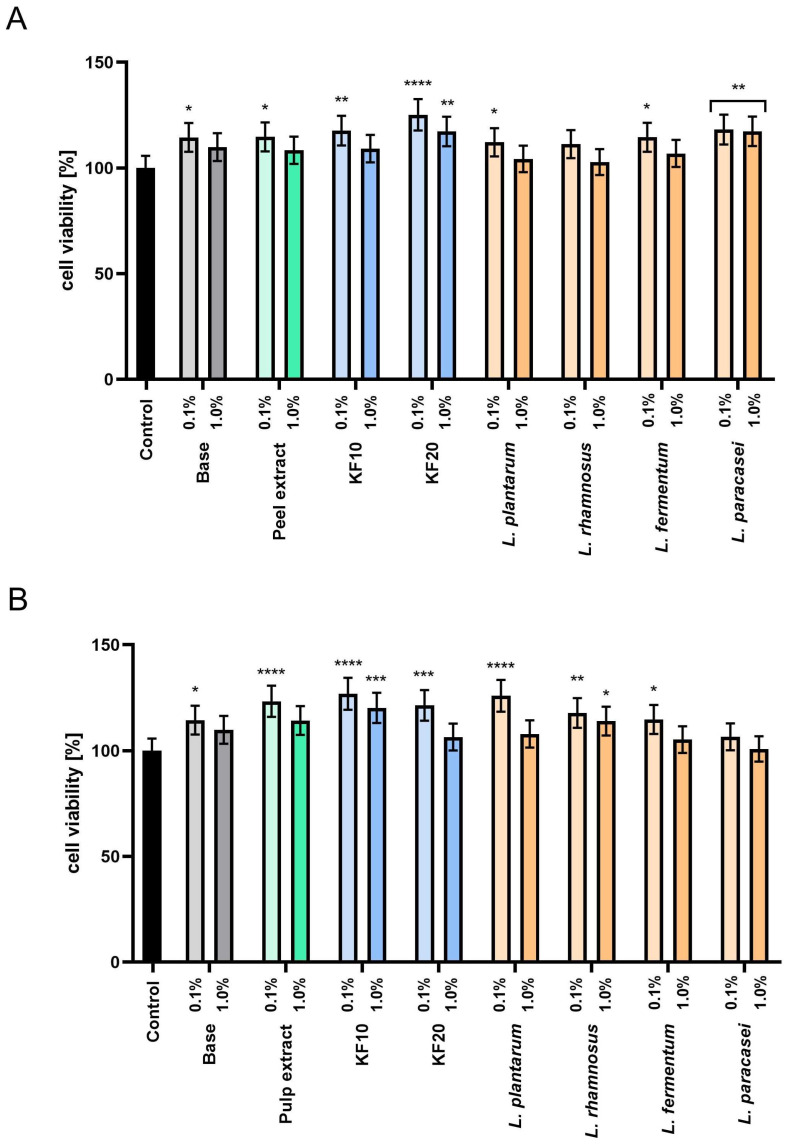
Assessment of neutral red uptake in keratinocytes (HaCaT) by moisturizing skin toners containing *C. pepo* L. peel (**A**) and pulp (**B**) extract and ferments at concentrations of 0.1 and 1.0%. Control cells without the addition of test samples, for which viability was assumed to be 100%. **** *p* < 0.0001, *** *p* < 0.001, ** *p* < 0.01, * *p* < 0.05.

The assessment of proliferation and cell metabolism using the Neutral Red test (Figure 7) confirmed the absence of cytotoxicity of all tested preparations. Furthermore, for *C. pepo* L. peel extracts and ferments (Figure 7A), the most beneficial effect was found for the kombucha ferment KF20 at a concentration of 0.1%, which increased cell viability to 125.14% ± 0.10. In the case of pumpkin pulp (Figure 7B), higher proliferation values were recorded in most samples, and the most beneficial effect was observed for the extract, kombucha ferments, and *L. plantarum* bacteria at a concentration of 0.1%. These results indicate that the pumpkin extracts and enzymes studied may be valuable raw materials with potential applications in cosmetic formulations that support the skin barrier function.

### 2.3. Assessment of Antioxidant Activity

#### 2.3.1. DPPH and ABTS Radical Scavenging

It is crucial to accurately assess the antioxidant properties of plant-derived substances, especially those that have undergone fermentation. Fermentation can increase the biological activity of raw materials by releasing bound phenolic compounds, producing new bioactive metabolites, and improving their bioavailability, which can significantly increase their effectiveness in protecting skin cells from oxidative stress. The presented analysis examined the antioxidant activity of pumpkin peel and pulp and its fermented counterparts (bioferments), obtained through fermentation using a culture of symbiotic microorganisms (kombucha) and the bacteria *L. plantarum*, *L. rhamnosus*, *L. fermentum*, and *L. paracasei*.

The DPPH and ABTS methods were used to determine antioxidant capacity, based on measuring the ability of the tested samples to neutralize synthetic free radicals. Both DPPH (2,2-diphenyl-1-picrylhydrazyl) and ABTS (2,2′-azino-bis (3-ethylbenzothiazoline-6-sulfonate) exhibit intense color, which is attenuated in the presence of reducing compounds, allowing for their quantitative determination using spectrophotometry.

**Figure 8:**
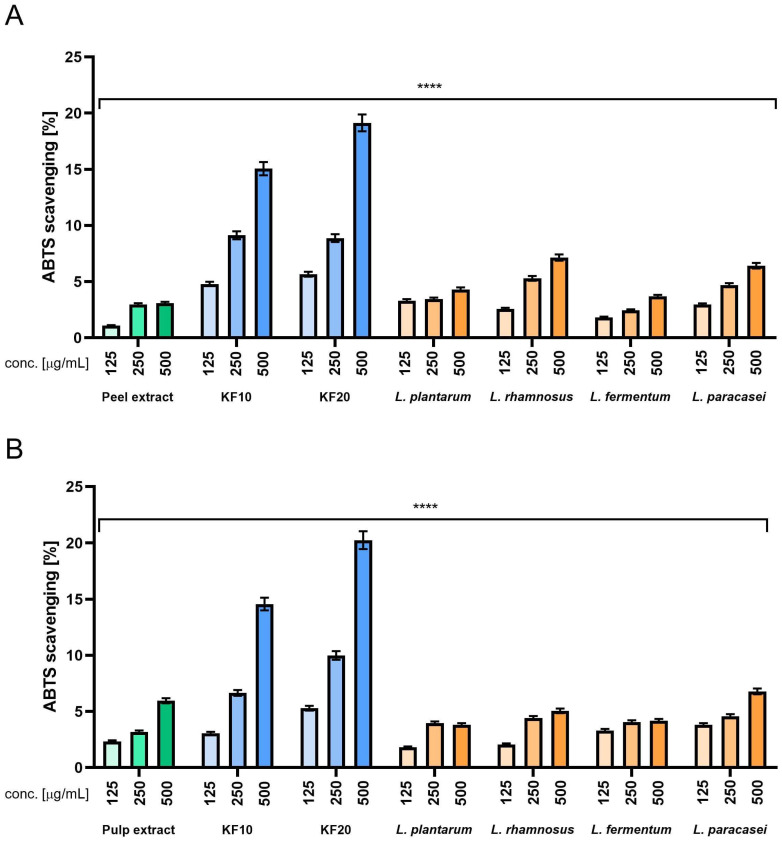
The ability of *C. pepo* L. peel (**A**) and pulp (**B**) extract and ferments to scavenge ABTS (2,2′-azino-bis-(3-ethylbenzothiazoline-6-sulfonic) acid) free radicals at concentrations of 125, 250, and 500 µg/mL. Ascorbic acid (125 µg/mL) and Trolox (125 µg/mL) were used as reference compounds. Data are presented as the mean ± standard deviation of three independent experiments, with each sample tested in triplicate. **** *p* < 0.0001.

The results obtained in ABTS experiments (Figure 8) allow us to formulate the following conclusions. First, in most cases, increased concentration resulted in increased scavenging effect. The one exception from this tendency is in *L. plantarum* (Figure 8B), but the drop observed between concentrations of 250 and 500 µg/mL is very small. Another observation is that when analyzing various variants of bacterial ferments (Figure 8A), we can see that the results are roughly twice those of the peel extract, while it has been shown in Figure 8B, most of the results obtained for bacterial ferments are on a comparable level with the pulp extract, or in some cases, even lower. When focusing on kombucha ferments, the overall result is very close to the previous experiment, as scavenging ability is noticeably higher compared to the peel (A) and pulp (B) extracts, with the results again being four to five times higher than for the extracts. Comparison between KF10 and KF20 indicates that KF20 performs better in all the concentrations that were tested. The best results across all the samples were observed for KF20 in the highest concentration of 500 µg/mL (Figure 8). Ascorbic acid and Trolox were used as reference samples at concentrations of 125 µg/mL, obtaining 92% and 96%, respectively.

**Figure 9:**
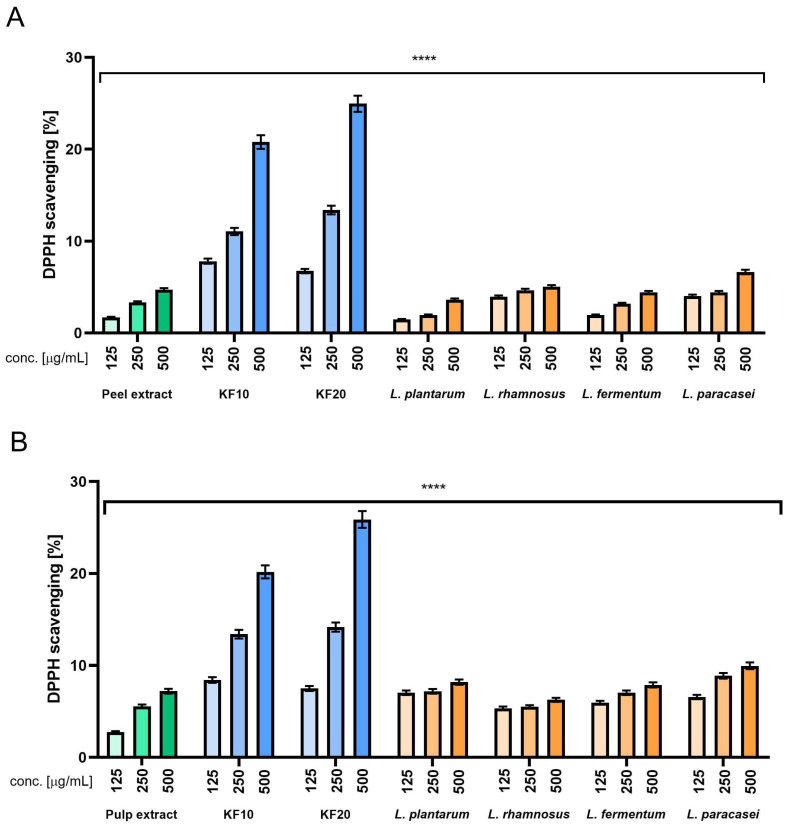
The ability of *C. pepo* L. peel (**A**) and pulp (**B**) extract and ferments to scavenge DPPH (2,2-diphenyl-1-picrylhydrazyl) free radicals at concentrations of 125, 250, and 500 µg/mL. Ascorbic acid (125 µg/mL) and Trolox (125 µg/mL) were used as reference compounds. Data are presented as the mean ± standard deviation of three independent experiments, with each sample tested in triplicate. **** *p* < 0.0001.

The results obtained in the DPPH test (Figure 9) allow us to formulate the following conclusions. First, for each sample increased concentration resulted in an increased scavenging effect. Another is that all the variants of bacterial ferments gave results comparable to the peel extract (Figure 9A) or just slightly higher compared to the pulp extract (Figure 9B). On the other hand, KF10 and KF20 ferments showed significantly higher scavenging effect, especially in the highest tested concentration of 500 µg/mL, with the scavenging result remaining even four or five times higher compared to all the other samples. Comparison between KF10 and KF20 depends on the concentration, as in the lowest concentration, KF10 performed more favorably, but in the moderate and the highest concentration tested, KF20 gave noticeably more beneficial results, the best among all the samples tested. Ascorbic acid and Trolox were used as reference samples at concentrations of 125 µg/mL, obtaining 88% and 94%, respectively.

#### 2.3.2. Intracellular ROS Levels in Fibroblasts and Keratinocytes

Additionally, the level of intracellular reactive oxygen species (ROS) generation was assessed using two types of skin cell lines: fibroblasts (HDF) and keratinocytes (HaCaT). The dye H_2_DCFDA (dichlorodihydrofluorescein-diacetate) was used for this purpose. The intensity of emitted fluorescence correlates with the concentration of reactive oxygen species and provides a measure of oxidative stress at the cellular level. To determine the cell response to oxidative stress, hydrogen peroxide (H_2_O_2_) was used at a concentration of 1 mM as a positive control, treating reference cells with it. H_2_O_2_ exposure induces an increase in ROS levels and thus provides a reference point for assessing the potential protective properties of the analyzed substances.

**Figure 10:**
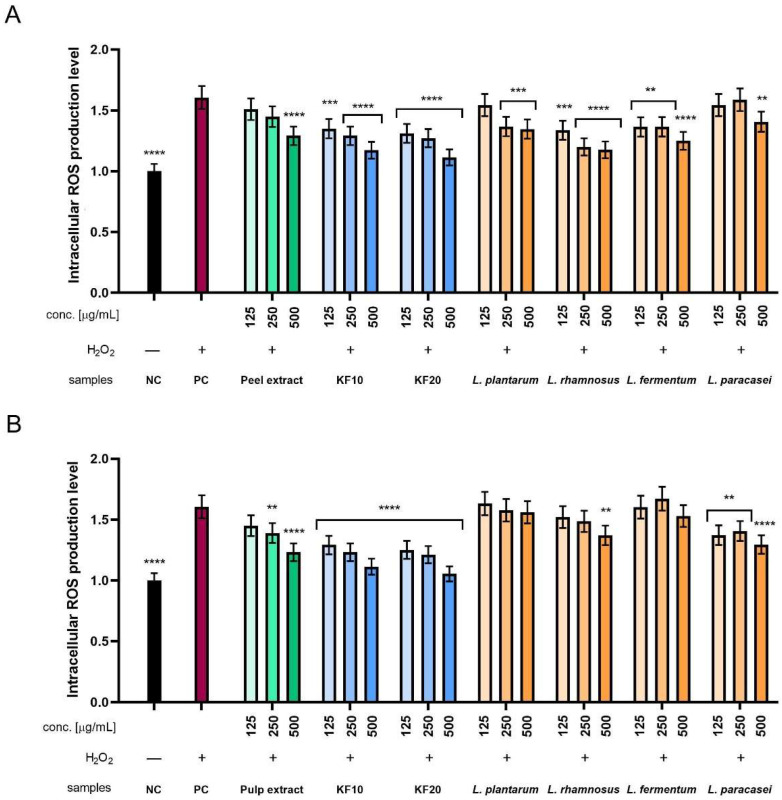
The effect of *C. pepo* L. peel (**A**) and pulp (**B**) extract and ferments at the concentrations of 125, 250, and 500 µg/mL on the intracellular level of reactive oxygen species in fibroblasts (HDFs). Data are presented as mean ± SD from three independent experiments, with each sample tested in triplicate. **** *p* < 0.0001, *** *p* < 0.001, ** *p* < 0.01.

In the case of ROS level analysis in HDF cells, there is a clear tendency for fluorescence to decrease with increasing concentration for all Kombucha ferments, peel extract, and several bacterial ferments. The values obtained for Kombucha ferments were more favorable compared to peel extract, where KF20 showed better antioxidant properties than KF10. The results obtained for *L. pantarum* and *L. paracasei* were comparable to the peel extract, but for the remaining *L. rhamnosus* and *L. fermentum*, they were comparable to kombucha. The most beneficial results among all tested samples were obtained for KF20 at the highest concentration of 500 µg/mL (Figure 10A). In the case of ROS level analysis for pumpkin pulp (Figure 10B), all bacterial ferments achieved less significant results compared to kombucha ferments. Only *L. paracasei* achieved results similar to those of the pulp extract. The best results among all tested samples were obtained for KF20 at the highest concentration of 500 µg/mL.

**Figure 11:**
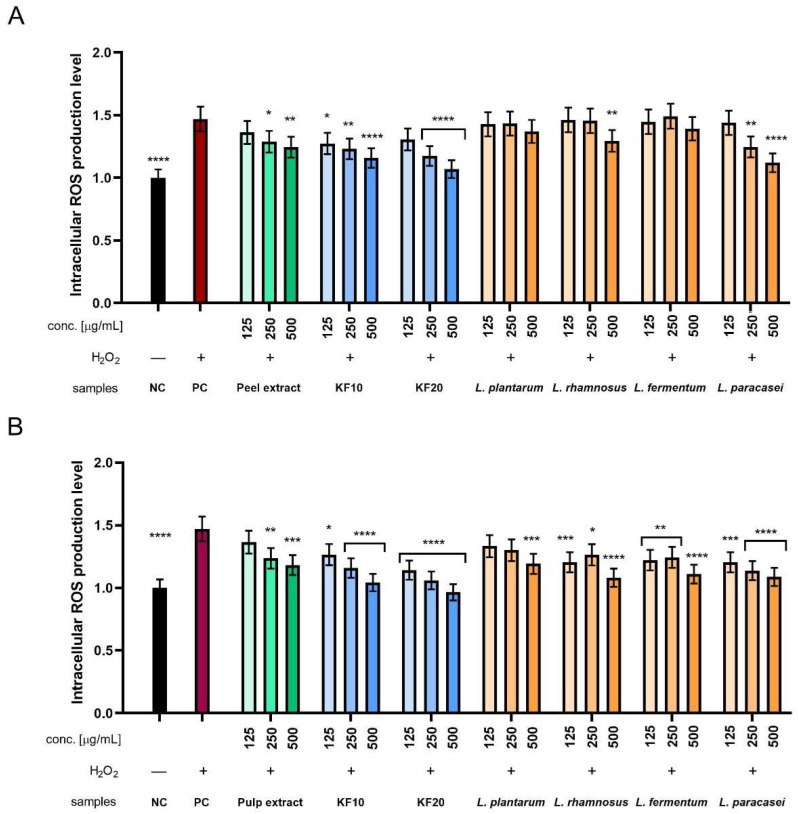
The effect of *C. pepo* L. peel (**A**) and pulp (**B**) extract and ferments at the concentrations of 125, 250, and 500 µg/mL on the intracellular level of reactive oxygen species in keratinocytes (HaCaTs). Data are presented as mean ± SD from three independent experiments, with each sample tested in triplicate. **** *p* < 0.0001, *** *p* < 0.001, ** *p* < 0.01, * *p* < 0.05.

When analyzing ROS levels in HaCaT cells, there is a clear trend that for each sample with Kombucha ferments and peel extract, normalized fluorescence decreases with concentration. This also applies to several bacterial ferment samples. In the case of KF10 and KF20, the decrease in fluorescence is significant and remains stronger compared to the peel extract. A decrease was also observed in the case of *L. paracasei*, where the values determined at the highest concentration of 500 µg/mL are only slightly higher compared to KF20 at the same concentration. The most favorable results among all tested samples were obtained for the KF20 ferment (Figure 11A). In the case of the analysis of antioxidant properties for the extract and enzymes from pumpkin pulp, it was observed that kombucha ferments achieved more favorable results than lactic acid bacterial ferments. As in the experiment with pumpkin peel, the most significant results among all the samples tested were obtained for the KF20 ferment. Furthermore, the results obtained for *L. plantarum* were similar to those for the pulp extract, but the other three showed slightly more beneficial performance.

### 2.4. Assessment of Extracellular Matrix (ECM) Degrading Enzymes Activity

As part of the study on ferments from *C. pepo* L. extract, the level of inhibition of enzymes responsible for skin aging was assessed. As it turned out, extracts and ferments from pumpkin peel and pulp exhibit significant anti-aging effects, resulting, among other things, from their ability to inhibit the activity of enzymes that degrade components of the skin’s extracellular matrix—such as collagenase, elastase, and hyaluronidase. These enzymes are responsible for the breakdown of collagen, elastin, and hyaluronic acid, and their excessive activity contributes to a decrease in skin elasticity, a reduction in its firmness, and wrinkle formation.

**Figure 12:**
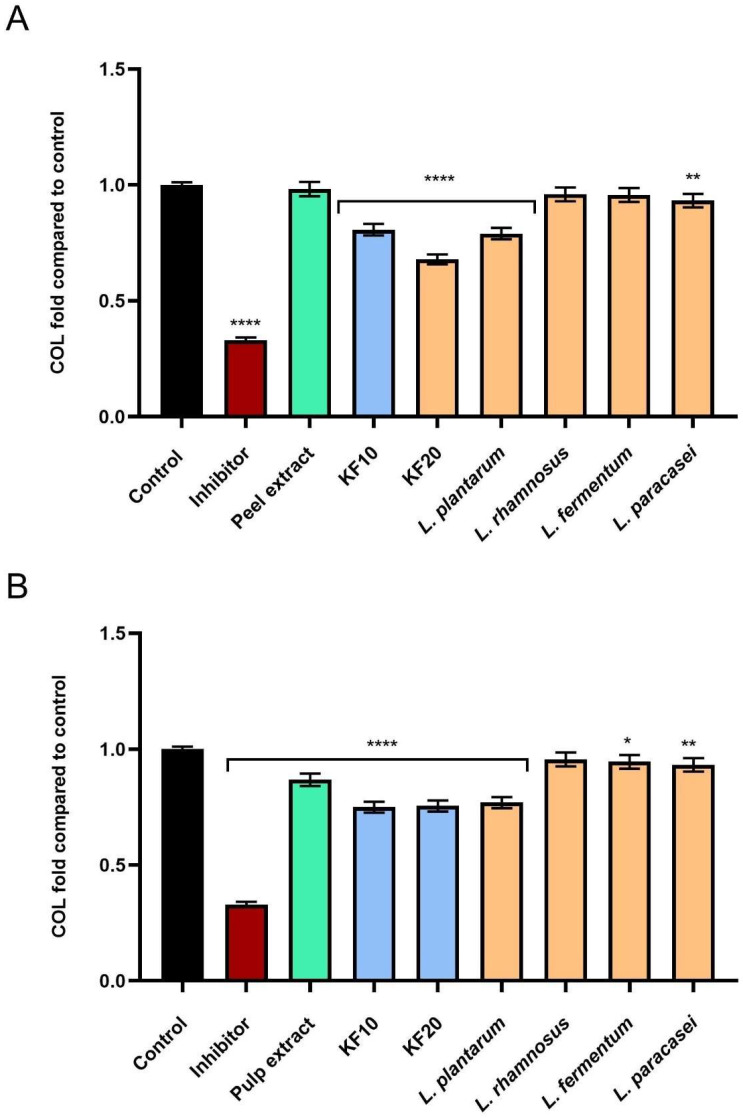
The impact of *C. pepo* L. peel (**A**) and pulp (**B**) extract and ferments on the level of collagenase (COL) (at the concentration of 250 µg/mL) calculated as a fold in comparison to the control. 1,10-phenanthroline (300 µM) was used as a reference control for collagenase inhibition. Data are mean ± SD from three independent experiments, in which each sample was tested in duplicate. **** *p* < 0.0001, ** *p* < 0.01, * *p* = 0.0115.

As shown in Figure 12A, the strongest inhibition of collagenase activity was demonstrated by the kombucha ferment *C. pepo* L. peel after 20 days of fermentation (KF20), reaching 0.674 ± 0.03-fold compared to the negative control (NC). In the case of pumpkin pulp (Figure 12B), the highest anti-aging activity was recorded for KF10, KF20, and the bacterial ferment *L. plantarum*, which reached 0.750 ± 0.04, 0.755 ± 0.05, and 0.770 ± 0.05-fold compared to the control, respectively.

**Figure 13:**
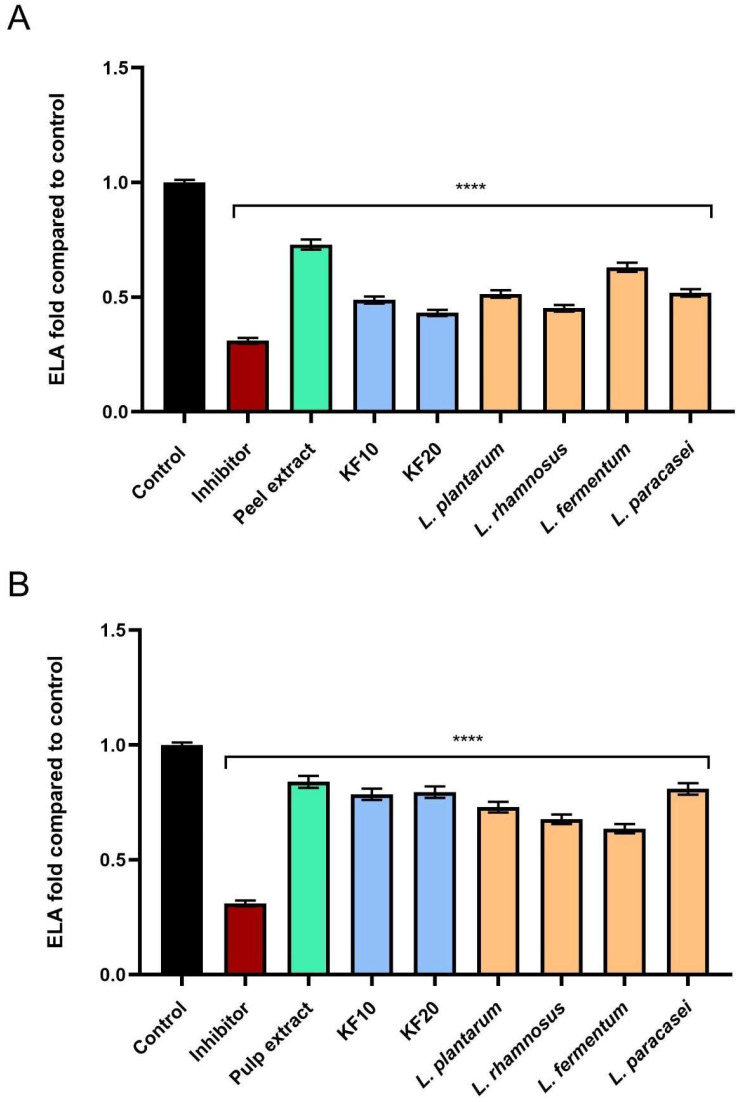
The impact of *C. pepo* L. peel (**A**) and pulp (**B**) extract and ferments on the level of elastase (ELA) (at the concentration of 250 µg/mL) calculated as a fold in comparison to the control. Succinyl-alanyl-alanyl-prolyl-valyl chloromethyl ketone (SPCK, 30 µM) was used as a referencecontrol for elastase inhibition. Data are mean ± SD from three independent experiments, in which each sample was tested in duplicate. **** *p* < 0.0001.

As shown in Figure 13, the tested samples also showed the ability to inhibit elastase activity, with particularly significant values obtained for kombucha and bacterial ferments. Pumpkin peel extract and ferments (Figure 13A) were characterized by higher levels of inhibition, and the most significant effects were obtained for KF10, KF20, and *L. rhamnosus*, which achieved 0.488 ± 0.03-, 0.432 ± 0.02-, and 0.451 ± 0.02-fold compared to the control, respectively.

**Figure 14:**
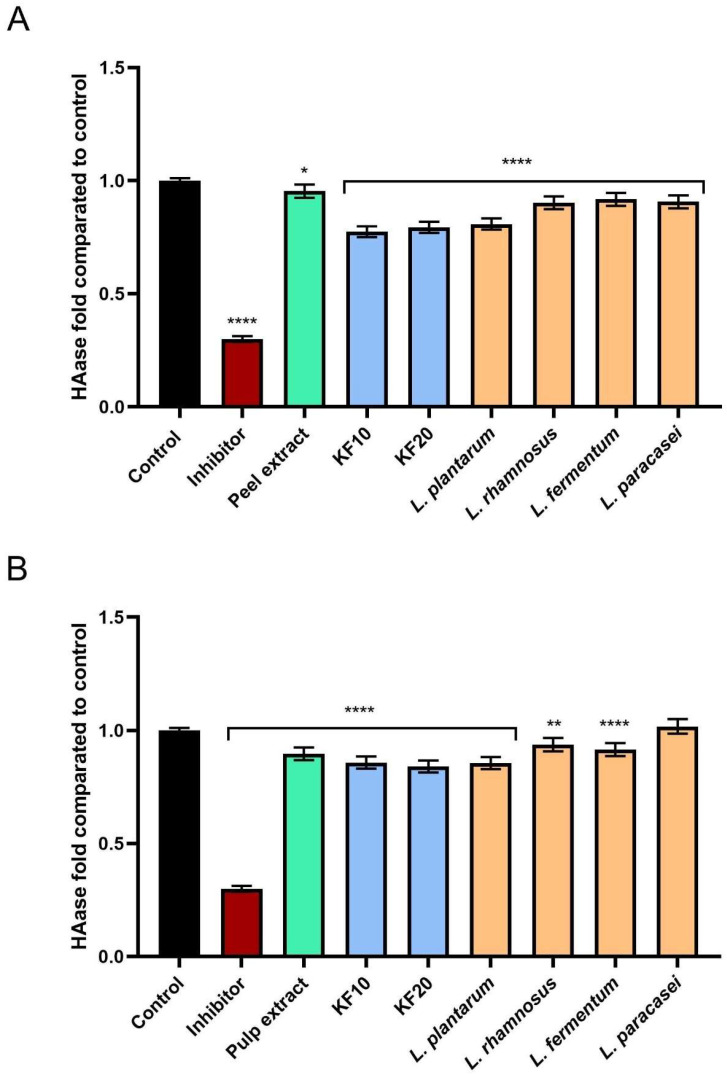
The impact of *C. pepo* L. peel (**A**) and pulp (**B**) extract and ferments on the level of hyaluronidase (HAase) (at the concentration of 250 µg/mL) calculated as a fold in comparison to the control. Tannic acid (300 µM) was used as a reference control for hyaluronidase inhibition. Data are mean ± SD from three independent experiments, in which each sample was tested in duplicate. **** *p* < 0.0001, ** *p* = 0.0027, * *p* = 0.0377.

Inhibition of hyaluronidase activity was noted in all analyzed samples (Figure 14). A slightly higher level of inhibition of this enzyme compared to the other samples was obtained for kombucha ferments (KF10 and KF20) from both pumpkin peel and pulp (Figure 14A,B). Among the lactic bacterial ferments, the most favorable results were obtained for *L. plantarum*, achieving 0.808 ± 0.05- and 0.855 ± 0.04-fold compared to the control, for pumpkin peel and pulp, respectively.

### 2.5. Assessment of Anti-Inflammatory Activity

This study assessed the effect of *C. pepo* L. peel and pump extracts and ferments on the expression of proinflammatory cytokines IL-6 and IL-1β in fibroblasts (HDF), which play a key role in the skin’s inflammatory response and regenerative processes. IL-6 is involved in activating the JAK/STAT pathway and promoting chronic inflammation, while IL-1β plays a role in inducing the acute phase reaction and activating other inflammatory mediators, such as TNF-α. Reducing the expression of these cytokines in fibroblasts may indicate the potential use of pumpkin extracts and ferments as ingredients in cosmetics and therapies supporting the treatment of skin inflammation.

*C. pepo* L. extracts and ferments were tested at a concentration of 250 µg/mL. All samples, including the positive control (PC), were treated with bacterial lipopolysaccharide (LPS) at a concentration of 10 µg/mL. Results are presented as fold change relative to the negative control NC (cells untreated with either the test compounds or LPS).

**Figure 15:**
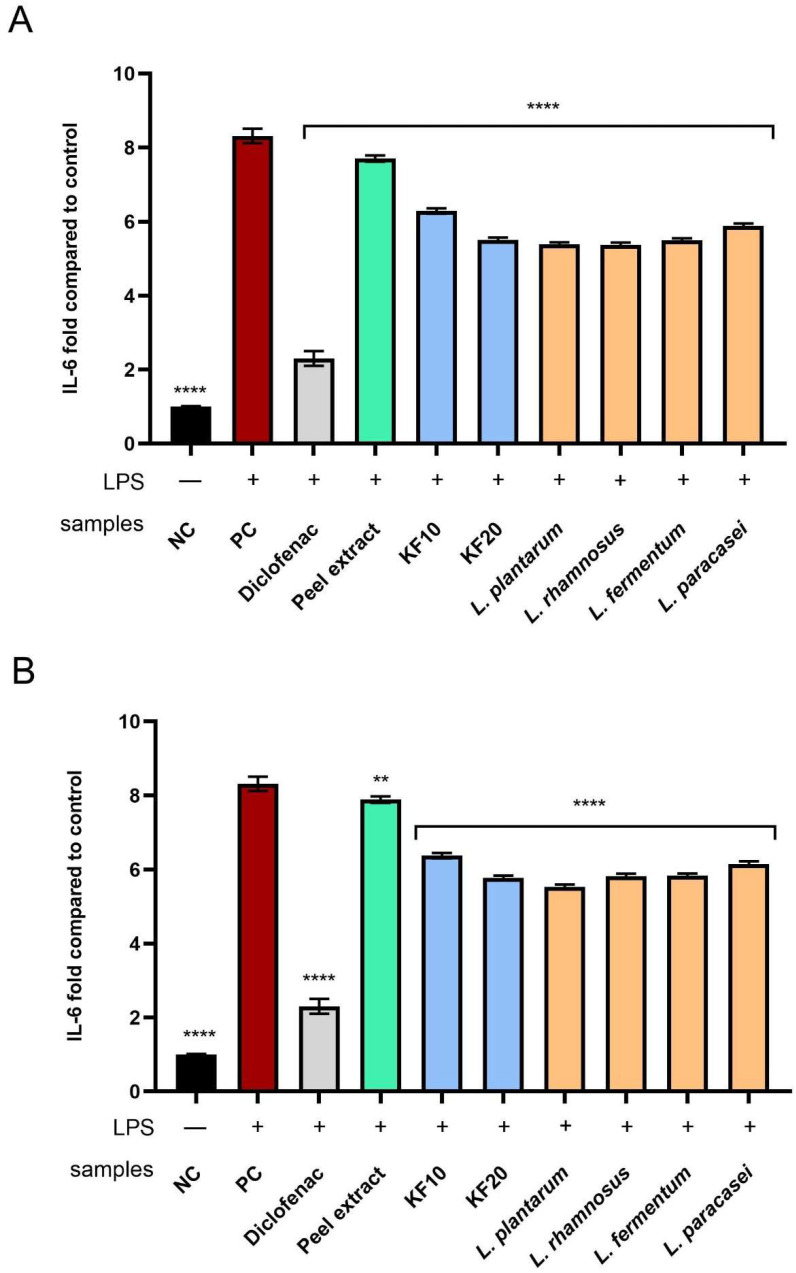
The effect of *C. pepo* L. peel (**A**) and pulp (**B**) extract and ferments (at the concentration of 250 µg/mL) after exposure to bacterial LPS (10 μg/mL) on the level of interleukin 6, calculated as a fold in comparison with the untreated control. Diclofenac was used as a reference compound. Data are mean ± SD from three independent experiments in which each sample was tested in duplicate. **** *p* < 0.0001, ** *p* = 0.0013.

As shown in Figure 15, all tested extracts and ferments effectively inhibited IL-6 activity. For both peel (Figure 15A) and pulp (Figure 15B), a stronger impact was observed for ferments—both those obtained with kombucha and *Lactobacillus* strains—compared to their respective extracts. In the case of *C. pepo* L. peel, the highest level of IL-6 inhibition was recorded for kombucha fermentation after 20 days (KF20), *L. plantarum*, and *L. rhamnosus*, reaching 5.51 ± 0.05-, 5.37 ± 0.03-, and 5.37 ± 0.05-fold to the control, respectively. However, in the case of *C. pepo* L. pulp, *L. plantarum* ferment showed the strongest anti-inflammatory effect, reaching 5.53 ± 0.04-fold compared to the NC.

**Figure 16:**
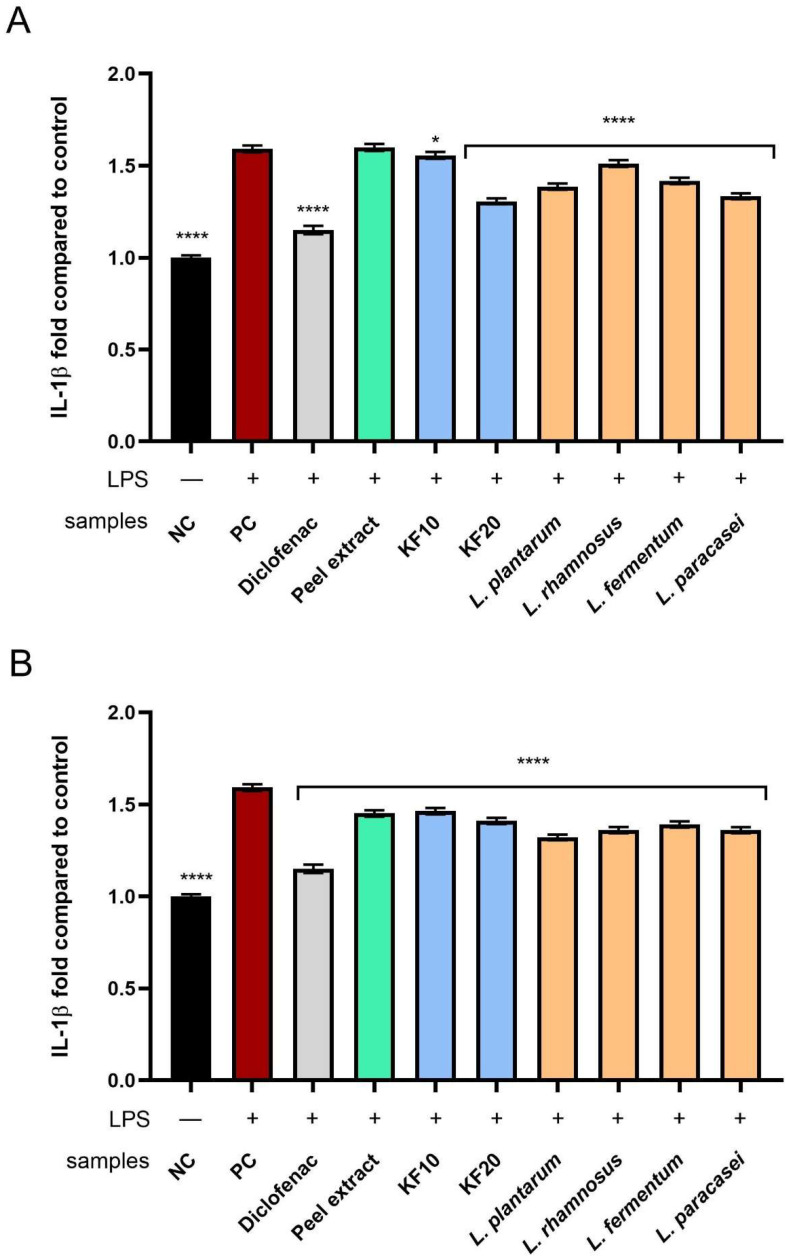
The effect of *C. pepo* L. peel (**A**) and pulp (**B**) extract and ferments (at the concentration of 250 µg/mL) after exposure to bacterial LPS (10 μg/mL) on the level of interleukin 1β, calculated as a fold in comparison with the untreated control. Diclofenac was used as reference compound. Data are mean ± SD from three independent experiments in which each sample was tested in duplicate. **** *p* < 0.0001, * *p* = 0.0199.

Analysis of the effect of the tested samples on the level of IL-1β showed that fermented extracts (from both *C. pepo* L. peel and pulp) inhibited its activity more effectively than the corresponding non-fermented extracts (Figure 16). In the case of pumpkin peel (Figure 16A), the strongest anti-inflammatory effect was recorded for the kombucha ferment after 20 days (KF20), reaching 1.31 ± 0.02-fold change compared to the control. In the case of the pulp (Figure 16B), lactic acid bacterial ferments showed a slightly stronger effect, among which the most favorable influence was observed for *L. plantarum* (1.32 ± 0.01-fold change compared to the negative control).

## 3. Discussion

Both the peel and pulp of pumpkins are rich sources of compounds with antioxidant properties. Studies on ferments have demonstrated a significant ability to neutralize free radicals, which is closely related to the content of polyphenolic compounds such as flavonoids (apigenin, kaempferol, quercetin), phenolic acids (gallic acid, chlorogenic acids), and tannins (galloylquinic acids). Fermentation of these raw materials is particularly important, as it can significantly increase their antioxidant potential and their application value in cosmetic and dermatological preparations.

The fermentation process, conducted both with a symbiotic culture of bacteria and yeast (SCOBY) and selected strains of bacteria (e.g., *L. plantarum*, *L. rhamnosus*, *L. fermentum*, *L. paracasei*), results in intense biochemical transformations. Available studies show that fermentation lasting 10 or 20 days contributes to a significant increase in the content of biologically active phenolic compounds, which were previously present in difficult-to-digest forms, such as glycosides or esters. The action of enzymes from fermenting microorganisms leads to their hydrolysis and the release of free forms, characterized by higher antioxidant activity. Furthermore, ferments obtained using kombucha demonstrate a significantly increased ability to neutralize free radicals compared to unfermented extracts [1,20,21,22]. This is also confirmed by in vitro studies using keratinocyte and fibroblast cell lines, in which fermented plant extracts, similarly to blueberry ferments, significantly reduced intracellular levels of reactive oxygen species (ROS), especially under oxidative stress conditions. A reduction in ROS fluorescence was observed, suggesting cytoprotective effects and the potential to protect skin cells from oxidative damage [23]. The obtained ferments may constitute an innovative raw material with antioxidant potential that can be used in modern protective and skincare cosmetics.

The findings of this study clearly indicate that both peel and pulp extracts derived from *C. pepo* L. are biocompatible with human skin cells. No signs of cytotoxicity were observed in HaCaT keratinocytes or HDF fibroblasts at any of the tested concentrations. Furthermore, selected fermentation processes, particularly those involving kombucha (10-day fermentation), substantially enhanced cell viability, as reflected in both mitochondrial and lysosomal activity. These results suggest that fermentation not only preserves the cytocompatibility of pumpkin-derived materials but may even potentiate their bioactivity through the generation or transformation of beneficial compounds. The absence of cytotoxic effects from pumpkin peel extracts aligns with previous reports. Gaweł-Bęben et al. [3] demonstrated that ethanolic extracts of peels from different *Cucurbita maxima* and *C. moschata* cultivars did not reduce keratinocyte viability at concentrations up to 1000 µg/mL. Only at 2000 µg/mL did certain varieties exhibit moderate cytotoxicity, with a reduction in viable HaCaT cells by approximately 40% [3]. These findings support the safe use of pumpkin peel extracts in topical applications at concentrations relevant to cosmetic and dermatological formulations. In the present study, fermentation emerged as a critical factor enhancing the biological activity of the extracts. Kombucha fermentation for 10 days notably increased mitochondrial and lysosomal activity in both cell types, especially in keratinocytes. This is likely due to the breakdown of complex plant polymers into low-molecular-weight compounds such as phenolic acids, amino acids, and short-chain organic acids, which have been shown to exert antioxidant and pro-proliferative effects on skin cells [12,24]. The observed decline in activity following 20-day kombucha fermentation suggests that extended fermentation may lead to the degradation or depletion of key bioactive compounds, as previously noted in other plant fermentation studies [25]. Ferments obtained with lactic acid bacteria (LAB) also demonstrated favorable effects on cell viability, particularly those involving *L. paracasei*, *L. rhamnosus*, and *L. fermentum*. These strains are known to secrete bioactive metabolites such as exopolysaccharides, peptides, and lactic acid, which support keratinocyte differentiation and fibroblast proliferation [26,27]. In contrast, ferments prepared with *L. plantarum* showed concentration-dependent reductions in viability, especially at higher doses. This trend is consistent with reports suggesting that specific metabolites produced by *L. plantarum*—including organic acids and bacteriocins—may negatively influence cell survival at elevated concentrations [28,29].

Taken together, the results support the hypothesis that fermentation serves as an effective strategy to enhance the biological value of pumpkin-based ingredients. The stimulation of mitochondrial and lysosomal functions in skin cells following treatment with fermented extracts is of particular interest for cosmetic and dermatological applications aimed at promoting skin regeneration, barrier repair, and overall tissue vitality. Further investigations into the specific chemical transformations occurring during fermentation could help clarify the molecular basis of these effects. The results presented in this study indicate that fermentation can effectively enhance the biological activity of plant-based by-products such as pumpkin peel and pulp, supporting their application in sustainable cosmetic formulations with regenerative and protective functions. Research indicates that other parts of the pumpkin also have potential cosmetic properties. For example, pumpkin seed oil is characterized by its ability to absorb up to 22% of UVB radiation, acting as a natural sunscreen [30]. Its high content of tocopherols (vitamin E) and phytosterols makes it a valuable ingredient in skin care products for mature, dry, and sun-damaged skin [31]. Furthermore, pumpkin seed extract has been shown to have anti-aging effects on fibroblasts damaged by doxorubicin. In the tested model, the extract reduced signs of cellular aging (including SA-β-galactosidase activity) in a concentration-dependent manner without demonstrating cytotoxicity. Furthermore, in silico analysis suggests that tocopherol may interact with the CYP3A4 enzyme isoform, which may provide a potential protective mechanism against oxidative stress and cellular damage [32].

Studies also show that pumpkin peel is rich in phenolic acids, flavonoids, and amino acids, which support regeneration, hydration, and protection of the extracellular matrix. Pumpkin flesh provides carotenoids (lutein, β-carotene) and tocopherols, which act as powerful antioxidants and protect against UV radiation [33,34]. Current cosmetics containing fermentation products demonstrate anti-aging effects on skin tissue, primarily through mechanisms such as moisturizing and repairing the skin barrier function, replenishing collagen and elastin, and acting as antioxidants [35]. Clinical studies confirm that oral probiotics can improve skin hydration and increase its water content, which translates into wrinkle reduction. In a hairless mouse epidermal model, oral administration of *Lactobacillus plantarum* reduced UV-induced water loss [36]. In another study, epidermal cells were exposed to *L. plantarum* L-137, which resulted in increased production of hyaluronic acid (HA). The mechanism of this process was explained to be based on the stimulation of the cytokine IFN-γ, which, together with TNF-α, activates the NF-κB pathway, resulting in increased expression of the enzyme hyaluronan synthase in fibroblasts and, consequently, increased production of hyaluronic acid, which is crucial for maintaining skin hydration and elasticity [37].

Furthermore, younger women (around 20 years of age) have been shown to have higher levels of *L. plantarum* bacteria in their skin than women around 50. Extracellular vesicles (EVs) secreted by these bacteria have anti-aging properties. They act by inhibiting the activity of MMP-1 and elastase enzymes, which are responsible for the breakdown of collagen and elastin, improving skin elasticity and maintaining normal cell shape. Furthermore, EVs increase the expression of filaggrin, a protein essential for maintaining the integrity and function of the epidermal barrier, whose levels naturally decline with age [38,39]. As shown in previous studies, *C. pepo* L. extract applied topically and orally in rats with contact dermatitis and chronic stress demonstrated a significant reduction in TNF-α and IL-6 levels, both systemically and locally in the skin, which was accompanied by a reduction in iNOS and COX-2 [40,41]. In another study using a wound-healing model in rats subjected to chronic stress, pumpkin extract significantly accelerated tissue regeneration, along with a reduction in serum and wound tissue levels of IL-6 and TNF-α. Pumpkin’s main active ingredients, such as carotenoids (β-carotene, lutein), tocopherols, phytosterols, and phenolic compounds, are known for their anti-inflammatory properties. Their synergistic action inhibits attacks by blocking the NF-κB and MAPK pathways—key regulators of cytokine gene transcription [1,2]. Moreover, lactic acid bacteria, particularly strains such as *L. plantarum*, *Bifidobacterium*, and *Streptococcus thermophilus*, have demonstrated anti-inflammatory effects in various skin models. Small clinical studies indicate that topical probiotics can alleviate symptoms of acne vulgaris, rosacea, and atopic dermatitis, suggesting their potential to modulate the skin’s immune response. Proposed mechanisms include stimulation of regulatory T cells, induction of anti-inflammatory cytokines (e.g., IL-10), competition with pathogens for nutrients, inhibition of bacterial biofilm formation, and blockade of pathogen-binding sites [42]. *L. plantarum*, a Gram-positive probiotic, produces antimicrobial peptides and bioactive metabolites with potential anti-inflammatory and skin-protective effects. One study reported that *Lactobacillus* extract improved epidermal barrier function, reduced erythema, decreased the skin’s microbial load, and may help in managing mild acne lesions [43]. These findings demonstrate that fermented pumpkin extracts are promising multifunctional ingredients for cosmetic applications, offering protective, regenerative, and skin-rejuvenating effects. Further in vivo studies are warranted to confirm their efficacy in topical formulations.

## 4. Materials and Methods

### 4.1. Plant Material, Extraction and Fermentation Procedure

The plant material (*C. pepo* L.) was obtained from controlled organic plantations where no artificial fertilizers or pesticides were applied. To prepare the extract, 18 g of *C. pepo* L. peel and pulp and 600 mL of distilled water at room temperature were used. The extraction process was carried out using a magnetic stirrer for 24 h at room temperature. After this time, the extracts were ultrasonically treated for 0.5 h at room temperature. The extracts were filtered using a vacuum pump (Aga Labor, Warsaw, Poland). The obtained extracts were marked as peel extract and pulp extract.

Fermentation with kombucha was performed using a SCOBY culture, consisting primarily of acetic acid bacteria and yeast, obtained from a commercial supplier in Poland (Nasza Przyszłość, Tapin, Poland). For this purpose, 100 mL samples of each of the three extracts were poured into separate, sterile 800 mL beakers. The solutions were supplemented with sucrose and kombucha starter to a final concentration of 10% (*w*/*v*). Fermentation was conducted for 10 and 20 days, respectively, in separate beakers at room temperature (approximately 25 °C) and protected from light. Ferments collected after 10 and 20 days were designated KF10 and KF20, respectively.

Lactic acid bacteria fermentation was performed using the strains *L. plantarum*, *L. rhamnosus*, *L. fermentum*, and *L. paracasei*. The process was carried out in bacteriological tubes, to which 4 mL of extract and 300 μL of the appropriate bacterial strain suspension were added. Fermentation continued for three days, after which the samples were centrifuged (ThermoFisher Scientific, Waltham, MA, USA) and the resulting supernatant was decanted and preserved for further analysis. The obtained ferments were named according to the bacterial strains used in the fermentation process: *L. plantarum*, *L. rhamnosus*, *L. fermentum*, and *L. paracasei*.

### 4.2. Determination of Biologically Active Compounds

All standard was obtained from MedChemExpress (Monmouth Junction, NJ, USA) and from Sigma-Aldrich (St. Louis, MO, USA).

The analytes were separated using an ultra-high-performance liquid chromatography (UHPLC) system from the Infinity II Series, equipped with a DAD detector and an Agilent 6224 ESI/TOF mass detector (Agilent Technologies, Santa Clara, CA, USA). The chromatographic system utilized a Kinetex C18 reversed-phase column 100Å (Phenomenex, Torrance, CA, USA) with dimensions of 150 mm × 2.1 mm, and a particle size of 1.7 µm. The chromatographic conditions were described previously [44].

Chromatographic analysis was performed within the wavelength range of 200 to 600 nm. The MS parameters were as follows: drying gas temperature of 325 °C, flow rate of 8 L/min, nebulizer pressure of 30 psi, capillary voltage of 3500 V, fragmentor voltage of 220 V, and skimmer voltage of 65 V [45].

### 4.3. Cytotoxicity Assessment

The cytotoxic potential of aqueous extracts and fermented preparations derived from *C. pepo* L. peel and pulp was evaluated using two complementary colorimetric assays: Alamar Blue (resazurin reduction) and Neutral Red uptake. The applied procedure followed the methodology previously described in our earlier study [46], with minor modifications. The tested materials included non-fermented aqueous extracts of peel and pulp, kombucha-fermented extracts obtained after 10 and 20 days of fermentation, and samples fermented with individual strains of lactic acid bacteria (*Lactobacillus paracasei*, *L. rhamnosus*, *L. fermentum*, and *L. plantarum*). All fermentations were conducted under controlled conditions using previously prepared aqueous extracts as substrates.

HaCaT human keratinocytes and HDF human dermal fibroblasts were used as representative skin cell models. Both skin cell lines were sourced from CLS Cell Lines Service (Eppelheim, Germany). Cells were cultured in Dulbecco’s Modified Eagle’s Medium (DMEM, Biological Industries, Cromwell, CO, USA) supplemented with 10% fetal bovine serum (FBS, Genos, Łódź, Poland) and 1% penicillin–streptomycin (Thermo Fisher Scientific, Waltham, MA, USA), and maintained at 37 °C in a humidified atmosphere with 5% CO_2_. For cytotoxicity assays, cells were seeded into 96-well plates at a density of 1 × 10^4^ cells/well and allowed to adhere for 24 h. All tested samples were diluted in culture medium to achieve final concentrations ranging from 50 to 500 µg/mL. After 24 h of exposure to the test samples, cellular metabolic activity was determined using the Alamar Blue assay, applying a 60 µM resazurin solution. Subsequently, lysosomal integrity was evaluated using the Neutral Red (40 µg/mL) uptake assay. Following dye uptake, HaCaT and HDF cells were washed twice with phosphate-buffered saline (PBS, Genos, Lodz, Poland), and the incorporated dye was subsequently extracted using a destaining solution composed of ethanol, acetic acid, and water (50:1:49, (*v*/*v*/*v*)). Absorbance readings were obtained using a microplate reader (Thermo Fisher Scientific, Waltham, MA, USA) at appropriate wavelengths (570 nm for Alamar Blue and 540 nm for Neutral Red). All measurements were performed in triplicate and normalized to untreated control cells, which were assigned 100% viability.

### 4.4. Determination of Antioxidant Properties

#### 4.4.1. ABTS^+^ Scavenging Assay

Method used to assess antioxidant activity involved the application of an ABTS-based assay [47]. Initially, a solution was prepared by combining 7 mM ABTS with 2.4 mM potassium persulfate in a 1:1 volume ratio. This mixture was then left to stand at room temperature for a minimum of 14 h to allow for the formation of ABTS^+^ radicals. After incubation, the solution was diluted with methanol until the absorbance reached approximately 1.0 at a wavelength of 734 nm.

Following this, sample solutions at concentrations of 125, 250, and 500 µg/mL were prepared. Each sample (1 mL) was mixed with 1 mL of the prepared ABTS solution, and the absorbance was recorded at λ = 734 nm using an Aquamate Helion UV/VIS spectrophotometer (Thermo Fisher Scientific, Waltham, MA, USA). As a control, a mixture of 1 mL ABTS solution with 1 mL methanol was used. The ability to scavenge ABTS^+^ radicals was then calculated using the appropriate formula.

(1) $$\%\;ABTS\;scavenging=\left(1-\left(\frac{Abs\;sample}{Abs\;control}\right)\right)\times 100$$

#### 4.4.2. DPPH Radical Scavenging Assay

To evaluate the antioxidant properties of the tested ferments and extracts, the DPPH (1,1-diphenyl-2-picrylhydrazyl) radical assay was employed [48]. Samples were prepared at concentrations of 125, 250, and 500 µg/mL and dispensed into a 96-well microplate (100 µL per well). Subsequently, 100 µL of a 4 mM DPPH solution dissolved in methanol was added to each well, followed by thorough mixing. Absorbance readings at a wavelength of 517 nm were taken every 5 min over a 30-min period using a UV-Vis Filter Max λ = 5 spectrophotometer (Thermo Fisher Scientific, Waltham, MA, USA). A solution of DPPH (100 µL per well) and purified water (100 µL per well) served as the control. Ascorbic acid (125 µg/mL) and Trolox (25 µg/mL) were used as reference compounds. Each measurement was carried out in triplicate. The DPPH radical scavenging activity was then calculated based on the obtained absorbance values using the appropriate formula.

(2) $$\%\;DPPH\;scavenging=\frac{Abs\;control-Abs\;sample}{Abs\;control}\times 100$$

#### 4.4.3. Detection of Intracellular Levels of Reactive Oxygen Species (ROS)

To evaluate the ability of *C. pepo* L. peel and pulp extracts and ferments to reduce intracellular reactive oxygen species (ROS) levels in skin cells, the procedure described by Zagorska-Dziok et al. [49] was applied. The fluorogenic dye H_2_DCFDA served as the detection probe. Human dermal fibroblasts (HDF) and keratinocytes (HaCaT) (CLS Cell Lines Service (Eppelheim, Germany) were seeded in 96-well plates at a density of 1 × 10^4^ cells/well and cultured for 24 h. Subsequently, the culture medium (DMEM) was replaced with DMEM solutions containing the tested extracts or ferments at concentrations of 125, 250, and 500 µg/mL, followed by a further 24 h incubation. After this period, the medium was removed, and cells were treated with 10 μM H_2_DCFDA (Sigma-Aldrich, St. Louis, MO, USA) prepared in DMEM without FBS. Hydrogen peroxide (H_2_O_2_) was then added to reach a final concentration of 500 μM. Positive controls consisted of cells exposed to H_2_O_2_ alone, whereas negative controls received neither H_2_O_2_ nor test samples. Following 60 min of incubation, fluorescence was measured at λ = 485 nm (excitation) and λ = 530 nm (emission) using a microplate reader (FilterMax F5, Thermo Fisher Scientific, Waltham, MA, USA). All analyses were conducted in three independent experiments, with each sample tested in triplicate.

### 4.5. Assessment of Extracellular Matrix (ECM) Degrading Enzymes Activity [46]

The inhibitory potential of *C. pepo* L. peel and pulp extracts and their ferments against collagenase, hyaluronidase, and neutrophil elastase was assessed through spectrophotometric measurements using the human COL2 α 1 ELISA kit, human HAase ELISA kit, and human NE/ELA2 ELISA kit (Elabscience Biotechnology Inc., Houston, TX, USA), following the manufacturer’s protocols. Human fibroblasts were cultured and exposed to the extract and ferments at concentration of 250 µg/mL. After treatment, the cells were lysed, and the ELISA procedure was carried out, including antibody incubation, washing steps, detection with biotinylated antibodies and HRP conjugates, and absorbance reading at 450 nm with a microplate reader (Thermo Fisher Scientific, Waltham, MA, USA). 1,10-phenanthroline (300 µM) was used as a reference control for collagenase inhibition. Succinyl-alanyl-alanyl-prolyl-valyl chloromethyl ketone (SPCK, 30 µM) was used as a reference control for elastase inhibition, and tannic acid (300 µM) was used as a reference control for hyaluronidase inhibition. The results were reported as fold change relative to the control group (HDF cells without exposure to test substances).

### 4.6. Assessment of Anti-Inflammatory Activity Measurement

The anti-inflammatory potential of extracts and ferments obtained from the peel and pulp of *C. pepo* L. was assessed by quantifying IL-1β, IL-6, and IL-10 levels in fibroblasts (HDFs) stimulated with bacterial lipopolysaccharide (LPS, 10 µg/mL) from *Escherichia coli* O111:B4 for 24 h. In parallel, cells grown in 6-well plates were treated with the tested extracts and ferments at concentration of 250 ug/mL. Following 24 h of incubation with LPS and the respective *C. pepo* L. peel and pulp extracts and ferments, the DMEM medium was removed, wells were rinsed with PBS, and 150 µL of RIPA buffer was added to induce cell lysis. The lysates were analyzed using commercial ELISA kits (Elabscience Biotechnology Inc., Houston, TX, USA) in accordance with the manufacturer’s protocols. Absorbance was recorded at 450 nm using a microplate reader (FilterMax F5, Thermo Fisher Scientific, Waltham, MA, USA). Untreated cells served as the negative control, whereas cells exposed to LPS without extracts or ferments served as the positive control. Diclofenac was used as a reference compound.

### 4.7. Preparation of the Model Skin Toners

The final composition of the analyzed model moisturizing skin toners is presented in Table 2. Raw materials commonly used in the cosmetics industry were used to prepare the samples. A 500 g toner was prepared according to the following procedure: purified water was poured into a glass beaker, trehalose and niacinamide were dissolved, and the remaining ingredients were stirred with a mechanical stirrer (Chemland O2O) until completely dissolved. The remaining ingredients were then added. The resulting base toner was divided into 15 equal parts. One of these parts was the model base toner without the addition of extracts and ferments. Pumpkin peel and pulp extracts/ferments were added to the remaining samples at a concentration of 10 wt. % in each sample.

### 4.8. Statistical Analysis

All results are expressed as mean values ± standard deviation (SD) from three separate experiments. Statistical evaluation was carried out using a one-way analysis of variance (ANOVA) followed by Dunnett’s and Tukey’s post-hoc tests. Differences were considered statistically significant at **** *p* < 0.0001, *** *p* < 0.001, ** *p* < 0.01, and * *p* < 0.05 compared with the control group. Data analysis was performed with GraphPad Prism software, version 8.4.3 (GraphPad Software, Inc., San Diego, CA, USA).

## 5. Conclusions

The study demonstrated that fermentation of *C. pepo* L. peel and pulp with kombucha (SCOBY) and selected *Lactobacillus* strains (*L*. *plantarum*, *L*. *rhamnosus*, *L. fermentum*, *L. paracasei*) significantly improved their phytochemical profile and biological activity. LC-MS analysis revealed that fermentation increased the content of bioactive compounds, including phenolic acids, flavonoids, and catechin derivatives, many of which were released from their bound forms through microbial enzymatic activity. This enrichment translated into significant improvements in antioxidant capacity, with kombucha fermentation—particularly after 20 days—demonstrating the highest DPPH and ABTS radical scavenging activity and the strongest reduction in intracellular ROS levels in keratinocytes (HaCaT) and fibroblasts (HDF). Cytoprotective tests (Alamar Blue, Neutral Red) confirmed that most ferments maintained or increased skin cell viability, with *L. paracasei*, *L. rhamnosus*, and *L. fermentum* demonstrating the most beneficial effects. Kombucha ferments at selected concentrations also demonstrated significant cytoprotective benefits. Anti-aging tests revealed significant inhibition of collagenase, elastase, and hyaluronidase—enzymes responsible for extracellular matrix degradation—with kombucha ferments generally achieving the strongest effect. Furthermore, all fermented samples demonstrated anti-inflammatory potential by significantly reducing IL-6 and IL-1β expression in LPS-stimulated fibroblasts. A prototype moisturizing toner formulated with pumpkin extracts and ferments retained cytoprotective activity in keratinocytes compared to a base toner without the test samples, confirming its functional value. Overall, fermented *C. pepo* L. peel and pulp represent a sustainable, multifunctional source of cosmetic ingredients with antioxidant, anti-aging, and anti-inflammatory properties, supporting their use in advanced skincare formulas designed to protect, regenerate, and maintain healthy skin.

## Figures and Tables

**Table 1:** Results of the quantitative analysis (±SD) of polyphenols in fermented extracts from pumpkin peel and pulp.

Component	Peel [µg/g ± SD]		Pulp [µg/g ± SD]	
	KF10	KF20	KF10	KF20
Gallic acid	24.97 ± 1.78 ^c^	41.09 ± 2.36 ^b^	25.06 ± 2.08 ^c^	50.64 ± 3.63 ^a^
Galloylquinic acids ^1^	28.08 ± 1.91 ^c^	40.23 ± 2.79 ^b^	32.19 ± 1.19 ^c^	48.98 ± 2.91 ^a^
Gallocatechin	5.98 ± 0.37 ^c^	7.45 ± 0.41 ^b^	13.41 ± 1.03 ^a^	13.50 ± 1.01 ^a^
Chlorogenic acids	7.73 ± 0.41 ^c^	5.56 ± 0.33 ^d^	14.20 ± 0.92 ^a^	10.60 ± 0.67 ^b^
*p*-Coumaroylquinic acids ^2^	7.56 ± 0.48 ^b^	10.48 ± 0.87 ^a^	6.57 ± 0.42 ^b^	10.71 ± 0.83 ^a^
Epigallocatechin	13.16 ± 0.75 ^c^	17.42 ± 0.81 ^b^	25.08 ± 1.92 ^a^	25.95 ± 2.01 ^a^
Catechin	0.33 ± 0.02 ^b^	0.43 ± 0.03 ^b^	3.31 ± 0.21 ^a^	3.29 ± 0.25 ^a^
Epicatechin	19.34 ± 1.06 ^b^	28.11 ± 1.56 ^a^	17.60 ± 1.18 ^b,c^	16.93 ± 1.42 ^c^
Apigenin derivative *m*/*z*-H = 563 ^3^	12.36 ± 0.56 ^c^	15.62 ± 0.78 ^b^	12.47 ± 0.82 ^c^	18.51 ± 1.16 ^a^
Apigenin diglucosides ^3^	16.62 ± 0.87 ^b^	20.19 ± 1.05 ^a^	16.69 ± 0.78 ^b^	22.68 ± 0.99 ^a^
Quercetin derivatives *m*/*z*-H = 771 ^4^	16.40 ± 0.85 ^c^	23.22 ± 1.17 ^a^	21.61 ± 0.55 ^b^	30.62 ± 1.45 ^a^
Apigenin derivative *m*/*z*-H = 5 77 ^3^	2.89 ± 0.17 ^c^	3.21 ± 0.15 ^c^	8.17 ± 0.63 ^b^	11.35 ± 0.57 ^a^
Kaempferol derivative *m*/*z*-H = 739 ^5^	11.09 ± 0.86 ^b^	13.94 ± 0.92 ^a^	nd	nd
Quercetin 3-O-rutinoside	4.03 ± 0.27 ^b^	4.23 ± 0.19 ^b^	4.84 ± 0.22 ^a^	5.38 ± 0.31 ^a^
Kaempferol derivatives *m*/*z*-H = 755 ^5^	11.44 ± 0.76 ^d^	13.85 ± 0.86 ^c^	15.02 ± 0.63 ^b^	19.43 ± 0.65 ^a^
Kaempferol 3-O-rutinoside	1.41 ± 0.02 ^b^	1.48 ± 0.01 ^b^	4.23 ± 0.12 ^a^	4.37 ± 0.16 ^a^

KF10—kombucha fermentation for 10 days; KF20—kombucha fermentation for 20 days ^1^—quantification was based on calibration curve for gallic acid; ^2^—quantification was based on calibration curve for p-coumaric acid; ^3^—quantification was based on calibration curve for apigenin 7-glucoside, ^4^—quantification was based on calibration curve for rutoside, ^5^—quantification was based on calibration curve for kaempferol rutinoside; nd—not detected. Different lowercase letters (a–d) indicate significant differences (*p* < 0.05).

**Table 2:** Formulation of the analyzed model skin tonics.

INCI Name	Concentration [wt. %]
Aqua	82.7
Glycerin	2.0
Trehalose	1.5
Niacinamide	2.0
D-Panthenol	1.0
Extract/Ferment	10.0
Dehydroacetic Acid and Benzyl Alcohol	0.8

## Data Availability

Data is contained within the article.

## Supplementary Materials

The following supporting information can be downloaded at: https://www.mdpi.com/article/10.3390/molecules30204082/s1, Table S1. Mass data of polyphenols found in extracts (E) and fermented extracts (F) from pumpkin peel and pulp; Figure S1. Base peak chromatograms of unfermented extracts obtained from the pulp (red) and peel (green) of *Cucurbita pepo* L.; Figure S2. Base peak chromatogram and extracted ion chromatograms in the mass range specific for aglycones, including apigenin (red), quercetin (green), and kaempferol (blue); Figure S3. Representative UV-Vis spectra extracted from chromatographic peaks assigned to flavonoids; Figure S4. Comparison of BPC chromatograms of fermented extracts from pumpkin skin obtained using different microbial species; Figure S5. Extracted ion chromatograms in the mass range corresponding to chlorogenic acids from unfermented peel extract (green) and from extracts after fermentation (with *L. plantarum* blue) and *L. fermentum* (red); Figure S6. Extracted ion chromatograms illustrating changes in sucrose and gluconic acid.
