# Supplementary material for: Anti-Aging, Anti-Inflammatory, and Cytoprotective Properties of Lactobacillus- and Kombucha-Fermented C. pepo L. Peel and Pulp Extracts with Prototype Skin Toner Development
Source: Molecules. 2025 Oct 14;30(20):4082. doi: 10.3390/molecules30204082 (PMC12566415; doi:10.3390/molecules30204082)
Supplement: Supplementary file 1 [file molecules-30-04082-s001.zip › molecules-3849235-supplementary.pdf]

molecules

Article

Anti-Aging, Anti-Inflammatory, and Cytoprotective Properties of *Lactobacillus*- and Kombucha-Fermented *C. pepo* L. Peel and Pulp Extracts with Prototype Skin Toner Development

Aleksandra Ziemlewska [1,*], Zofia Nizioł-Łukaszewska [1], Martyna Zagórska-Dziok [1], Agnieszka Mokrzyńska [1], Witold Krupski [2], Magdalena Wójciak [3] and Ireneusz Sowa [3]

[1] Department of Technology of Cosmetic and Pharmaceutical Products, Medical College, University of Information Technology and Management in Rzeszow, Sucharskiego 2, 35-225 Rzeszow, Poland; zniziol@wsiz.edu.pl (Z.N.-Ł.); mzagorska@wsiz.edu.pl (M.Z.-D.); amokrzynska@wsiz.edu.pl (A.M.)
[2] II Department of Medical Radiology, Medical University of Lublin, Aleje Raclawickie 1, 20-059 Lublin, Poland; witold.krupski@umlub.edu.pl
[3] Department of Analytical Chemistry, Medical University of Lublin, Aleje Raclawickie 1, 20-059 Lublin, Poland; magdalena.wojciak@umlub.edu.pl (M.W.); ireneusz.sowa@umlub.edu.pl (I.S.)
* Correspondence: aziemlewska@wsiz.edu.pl

Table S1. Mass data of polyphenols found in extracts (E) and fermented extracts (F) from pumpkin peel and pulp.

R_T (min.)	Mass data (m/z-H)	Formula	Δ ppm	Component	Peel extract	Peel ferment	Pulp extract	Pulp ferment
4.76	169.01488	$C_7H_6O_5$	3.72	Gallic acid	-	+	-	+
5.95	343.06771	$C_{14}H_{16}O_{10}$	1.86	Galloylquinic acid	-	+	-	+
6.82	343.06743	$C_{14}H_{16}O_{10}$	1.05	Galloylquinic acid	-	-	-	+
9.38	305.06792	$C_{15}H_{14}O_7$	4.06	gallocatechin	-	+	-	+
11.22	353.08891	$C_{16}H_{18}O_9$	3.12	Neochlorogenic acid	+	+	+	+
13.32	337.09303	$C_{16}H_{18}O_8$	0.41	3-p-Coumaroylquinic acid	-	+	-	+
14.10	337.09329	$C_{16}H_{18}O_8$	1.18	3-p-Coumaroylquinic acid	-	+	-	+
14.31	305.06704	$C_{15}H_{14}O_7$	1.19	Epigallocatechin	-	+	-	+
15.56	289.07201	$C_{15}H_{14}O_6$	0.86	Catechin	-	+	-	+
16.30	353.08889	$C_{16}H_{18}O_9$	3.06	Chlorogenic acid	+	+	+	+
17.75	337.09349	$C_{16}H_{18}O_8$	1.77	4-p-Coumaroylquinic acid	-	+	-	+
19.49	289.07192	$C_{15}H_{14}O_6$	0.55	Epicatechin	-	+	-	+
19.65	337.09401	$C_{16}H_{18}O_8$	3.31	4-p-Coumaroylquinic acid	-	+	-	+
20.30	337.09369	$C_{16}H_{18}O_8$	2.36	5-p-Coumaroylquinic acid	-	+	-	+
20.94	593.14969	$C_{27}H_{30}O_{15}$	-2.53	Apigenin diglucoside	-	+	-	+
22.62	337.09411	$C_{16}H_{18}O_8$	3.61	5-p-Coumaroylquinic acid	-	+	-	+
24.73	563.14298	$C_{26}H_{28}O_{14}$	4.17	Apigenin derivative	-	+	-	+
27.71	771.19866	$C_{33}H_{40}O_{21}$	-0.35	Quercetin derivative	-	+	-	+
27.99	593.14975	$C_{27}H_{30}O_{15}$	-2.43	Apigenin diglucoside	-	+	-	+
29.53	577.15805	$C_{27}H_{30}O_{14}$	3.06	Apigenin derivative	-	+	-	+
29.87	771.19899	$C_{33}H_{40}O_{21}$	0.08	Quercetin derivative	-	+	-	+
30.92	577.15853	$C_{27}H_{30}O_{14}$	3.89	Apigenin derivative	-	+	-	+
31.05	739.20784	$C_{33}H_{40}O_{19}$	-1.71	Kaempferol derivative	-	+	-	-
31.56	755.20578	$C_{33}H_{40}O_{20}$	2.33	Kaempferol derivative	-	+	-	+
31.94	609.14711	$C_{27}H_{30}O_{16}$	1.64	Quercetin 3-O-rutinoside	-	+	-	+
35.52	755.20703	$C_{33}H_{40}O_{20}$	3.98	Kaempferol derivative	-	+	-	+

37.93	593.15043	$C_{27}H_{30}O_{15}$	-1.29	Kaempferol 3-O-rutinoside	-	+	-	+
44.99	677.49577<br>723 (m/z+HCOOH)	$C_{35}H_{70}N_2O_{10}$	3.24	unknown	+	+	+	+
68.14	286.23925	$C_{16}H_{33}NO_3$	1.68	unknown	+	+	+	+
69.41	293.17627	$C_{17}H_{26}O_4$	1.49	unknown	+	+	+	+

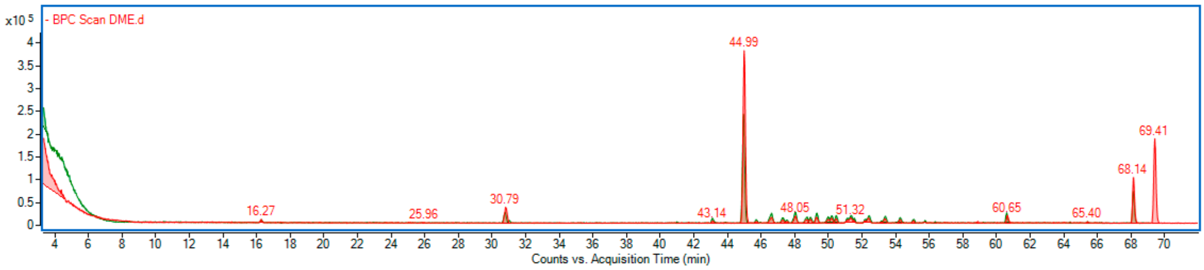

Figure S1. Base peak chromatograms of unfermented extracts obtained from the pulp (red) and peel (green) of *Cucurbita pepo* L.

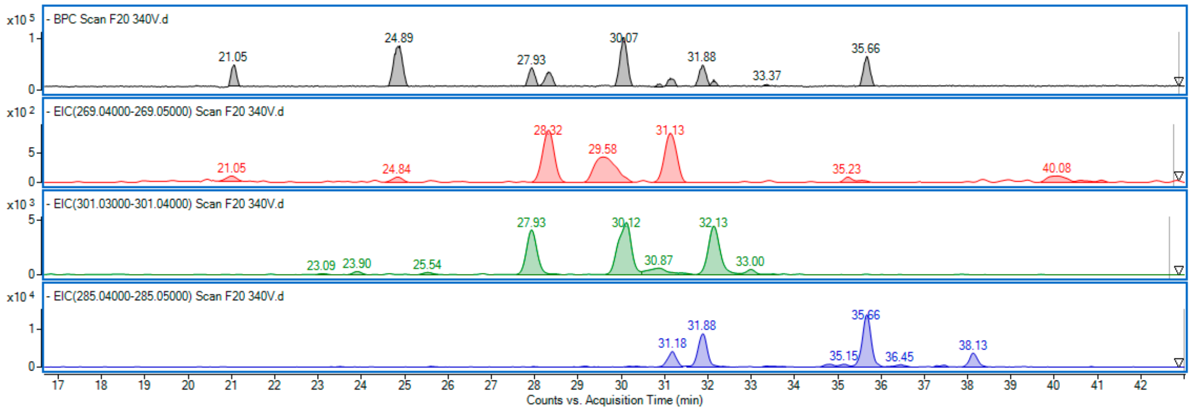

Figure S2. Base peak chromatogram and extracted ion chromatograms in the mass range specific for aglycones, including apigenin (red), quercetin (green), and kaempferol (blue).

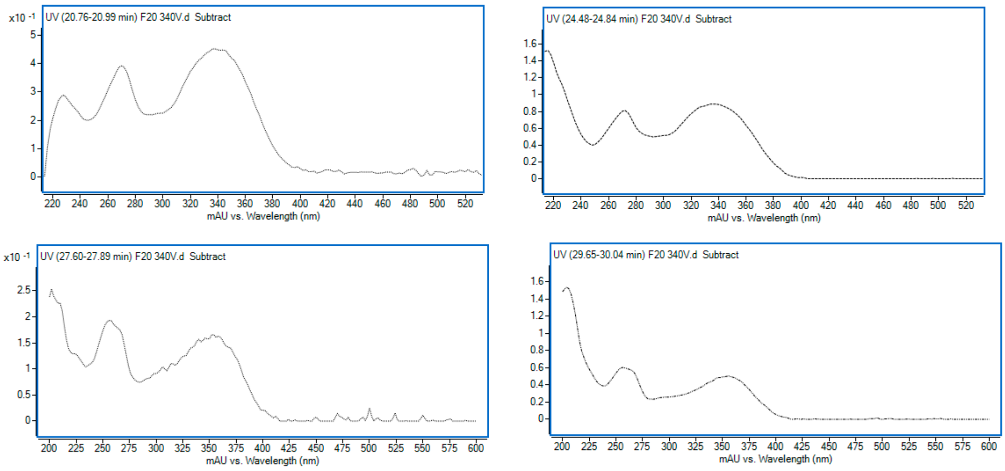

Figure S3. Representative UV-Vis spectra extracted from chromatographic peaks assigned to flavonoids.

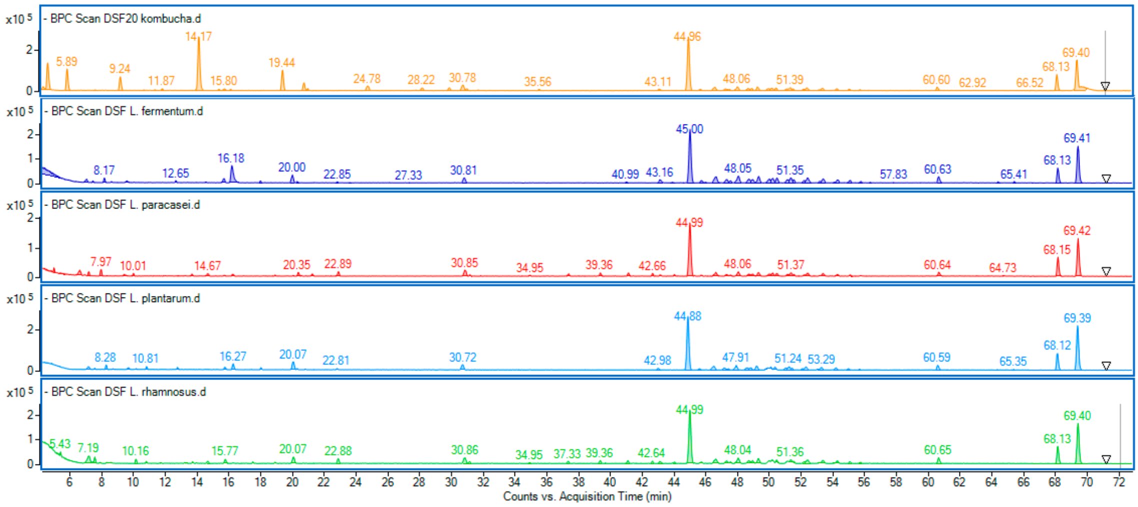

Figure S4. Comparison of BPC chromatograms of fermented extracts from pumpkin skin obtained using different microbial species.

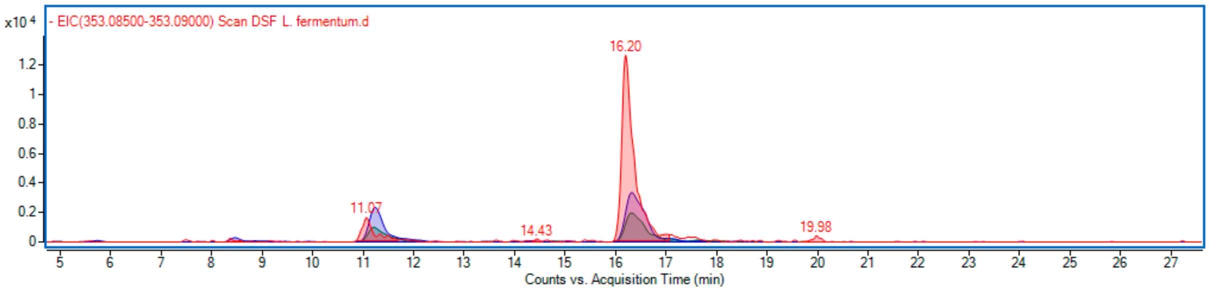

Figure S5. Extracted ion chromatograms in the mass range corresponding to chlorogenic acids from unfermented peel extract (green) and from extracts after fermentation (with *L. plantarum* blue) and *L. fermentum* (red).

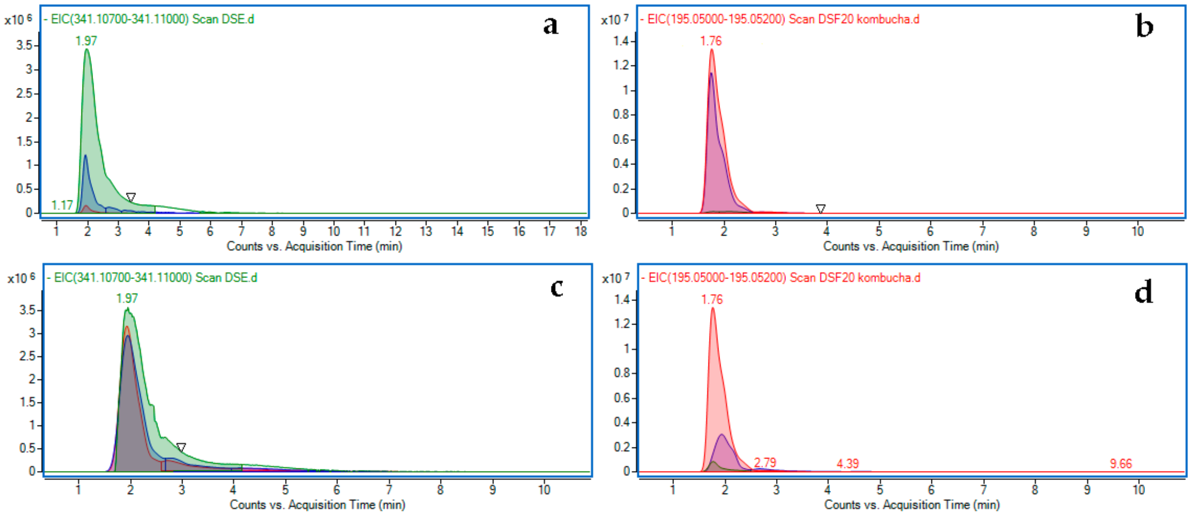

Figure S6. Extracted ion chromatograms illustrating changes in sucrose and gluconic acid. a - comparison of sucrose levels in the unfermented peel extract (green) and in extracts after 10 days (blue) and 20 days (red) of fermentation with kombucha; b - comparison of gluconic acid levels in the unfermented skin extract (green) and in extracts after 10 days (blue) and 20 days (red) of fermentation with kombucha; c - comparison of sucrose levels in the unfermented peel extract (green) and in extracts after fermentation with *L. plantarum* (red) and *L. fermentum* (blue); d - comparison of gluconic acid levels in extracts after fermentation with kombucha (red), *L. plantarum* (blue), and *L. fermentum* (green).
